# Selection of High Producers From Combinatorial Libraries for the Production of Recombinant Proteins in *Escherichia coli* and *Vibrio natriegens*

**DOI:** 10.3389/fbioe.2019.00254

**Published:** 2019-10-04

**Authors:** Joel Eichmann, Markus Oberpaul, Tobias Weidner, Doreen Gerlach, Peter Czermak

**Affiliations:** ^1^Institute of Bioprocess Engineering and Pharmaceutical Technology, University of Applied Sciences Mittelhessen, Giessen, Germany; ^2^Faculty of Biology and Chemistry, Justus-Liebig University of Giessen, Giessen, Germany; ^3^Branch for Bioresources, Fraunhofer Institute for Molecular Biology and Applied Ecology, Giessen, Germany

**Keywords:** Golden Gate, MoClo, protein secretion, genetic engineering, high-throughput screening, antimicrobial peptide, uricase

## Abstract

The optimization of recombinant protein production in bacteria is an important stage of process development, especially for difficult-to-express proteins that are particularly sensitive or recalcitrant. The optimal expression level must be neither too low, which would limit yields, nor too high, which would promote the formation of insoluble inclusion bodies. Expression can be optimized by testing different combinations of elements such as ribosome binding sites and N-terminal affinity tags, but the rate of protein synthesis is strongly dependent on mRNA secondary structures so the combined effects of these elements must be taken into account. This substantially increases the complexity of high-throughput expression screening. To address this limitation, we generated libraries of constructs systematically combining different ribosome binding sites, N-terminal affinity tags, and periplasmic translocation sequences representing two secretion pathways. Each construct also contained a green fluorescent protein (GFP) tag to allow the identification of high producers and a thrombin cleavage site enabling the removal of fusion tags. To achieve proof of principle, we generated libraries of 200 different combinations of elements for the expression of an antimicrobial peptide (AMPs), an antifungal peptide, and the enzyme urate oxidase (uricase) in *Escherichia coli* and *Vibrio natriegens*. High producers for all three difficult-to-express products were enriched by fluorescence-activated cell sorting. Our results indicated that the *E. coli* ssYahJ secretion signal is recognized in *V. natriegens* and efficiently mediates translocation to the periplasm. Our combinatorial library approach therefore allows the cross-species direct selection of high-producer clones for difficult-to-express proteins by systematically evaluating the combined impact of multiple construct elements.

## Introduction

*Escherichia coli* is one of the most common production hosts for recombinant proteins and many expression construct components are available to improve yields and facilitate protein recovery, including a variety of promoters and fusion proteins (Moore et al., 2016; Schreiber et al., 2017). Although, many recombinant proteins are strongly expressed with short production times and can be recovered as soluble products, others are difficult to express either because they are toxic to the host, resulting in low yields, or because they form insoluble inclusion bodies due to incomplete or incorrect folding (Carrió and Villaverde, 2002; Saïda, 2007). These challenges are often encountered when expressing antimicrobial peptides (AMPs), many of which require the formation of disulfide bonds for efficient folding and biological activity. In some cases inclusion bodies are produced deliberately, but the purification and refolding of insoluble proteins requires elaborate downstream processing and often limits the yield and functionality of the product (Hoffmann et al., 2018).

The folding of recombinant proteins in bacteria can also be improved by targeting the periplasm, a space between the outer and inner membrane that contains more chaperones than the cytoplasm, thus promoting folding and resulting in higher yields and less complex downstream processing steps. The oxidizing environment of the periplasm also favors the formation of disulfide bonds (Choi and Lee, 2004; Balasundaram et al., 2009). Translocation of proteins to the periplasm has been a subject to research since the very beginnings of recombinant protein expression technology (Gray et al., 1985; Oliveira et al., 1999). There are two major protein secretion pathways in bacteria. The general secretory (Sec) pathway exports unfolded proteins during or after translation (Tsirigotaki et al., 2017) whereas the twin-arginine translocation (Tat) pathway exports folded proteins. Secretion to the periplasm is achieved by adding an N-terminal signal peptide, which is cleaved off during translocation (Berks et al., 2000). Overexpression of the Tat machinery has been shown to increase the rate of recombinant protein secretion (Browning et al., 2017).

The optimization of recombinant protein production requires the appropriate combination of regulatory elements to control expression and fusion tags for purification. Translation initiation occurs at a ~35 bp sequence upstream of the start codon, containing the Shine-Dalgarno sequence. This so called ribosome binding site strongly effects the mRNA secondary structure (Dreyfus, 1988; Gold, 1988). Expression level screening is often based on combinatorial libraries of short constitutive promoters and/or ribosome binding sites (Coussement et al., 2014; Mahr et al., 2016). Multiple platforms for fusion tag screening have also been developed for bacteria, yeasts, and animal cells. However, all require the laborious cloning of large numbers of expression constructs (Abdulrahman et al., 2009; Sinah et al., 2012; Steinmetz and Auldridge, 2017).

One of the major drawbacks of high-throughput screening is that the expression levels and fusion tags are generally considered independently (one factor at a time), for example by using the same ribosome binding site for all constructs and testing a range of fusion tags. This is disadvantageous because the rate of protein synthesis strongly depends on mRNA secondary structures, so the combination of elements used in an expression construct can have a profound effect on its performance (Punginelli et al., 2004; Espah Borujeni and Salis, 2016). To overcome this challenge, we implemented a platform to generate combinatorial libraries of ribosome binding sites, secretion signals, and fusion tags. The library included five ribosome binding sites systematically combined with the Tat-specific signal peptides ssTorA and ssNapA, as well as the ssDmsA, ssYahJ, and ssYcdB peptides that are recognized in both the Tat and Sec pathways. All signal peptides have been previously demonstrated to mediate periplasmic translocation in *E. coli* (Tullman-Ercek et al., 2007; Fisher et al., 2008). These elements were further combined with four different N-terminal affinity tags for purification: glutathione-S-transferase (GST), maltose-binding protein (MBP), the small ubiquitin-like modifier (SUMO), and thioredoxin. All four tags have been shown to improve the solubility of recombinant proteins (Young et al., 2012), and affinity chromatography methods are available based, respectively, on immobilized glutathione (Schäfer et al., 2015), amylose (Reuten et al., 2016; Han et al., 2018), SUMO-specific antibodies (Butt et al., 2005), and the formation of reversible disulfide bonds (Mambetisaeva et al., 1997; McNiff et al., 2016). All four fusion tags were also combined with an additional N-terminal His_6_ tag for purification by immobilized metal affinity chromatography (IMAC) (Loughran et al., 2017), resulting in eight different purification tag versions in the library. By combing the five ribosome binding sites, five secretion signals, and eight purification tags, the library contained 200 combinations of elements in total.

Libraries were assembled for three difficult-to-express proteins. The first product was the insect metalloproteinase inhibitor (IMPI) from the greater wax moth *Galleria mellonella* (Wedde et al., 1998). This AMP is 69 amino acids in length and contains five disulfide bonds, a major challenge for bacterial expression systems. The production of soluble IMPI has been achieved in redox-engineered *E. coli* strains with an oxidizing cytoplasm that promotes disulfide bond formation (Joachim et al., 2019). Moreover, IMPI has been produced with high yields in the form of inclusion bodies, and was successfully resolubilized using the Cry4AaCter pull-down tag (Hoffmann et al., 2019). However, IMPI has yet to be expressed as a soluble product by targeting the periplasm. The second product was the antifungal peptide lucimycin from the common green bottle fly *Lucilia sericata*. This peptide is 77 amino acids in length and has been expressed as a soluble product in *E. coli*, but with low yields (Pöppel et al., 2014; Schreiber et al., 2017). The third product was the enzyme urate oxidase (uricase) also from *L. sericata*. This enzyme is localized in the Malpighian tubes (Baumann et al., 2017) and catalyzes the degradation of uric acid to 5-hydroxyisourate, which is then converted to allantoin (Ramazzina et al., 2006). It has previously been expressed in *E. coli* albeit in the form of inclusion bodies (Baumann et al., 2017). All three targets were expressed as fusion proteins containing a secretion signal, fusion tag, a green fluorescent protein (GFP) fusion partner to allow the identification of high-producer clones by fluorescence activated cell screening (FACS), and a thrombin recognition site to cleave the fusion partners from the target protein.

Although, the expression libraries were designed for *E. coli* strain BL21, we also used them to optimize recombinant protein expression in V_max_ Express, a commercial strain of the fast-growing γ-proteobacterium *Vibrio natriegens* engineered for protein production by integrating a T7 expression cassette (Weinstock et al., 2016). This bacterium can utilize inexpensive carbon sources and has a high growth rate, making it ideal for biotechnology applications (Hoffart et al., 2017). It was recently demonstrated to be an alternative host for recombinant protein production (Schleicher et al., 2018; Becker et al., 2019). Here, we report the successful transfer of multiple expression construct elements from *E. coli* to *V. natriegens*, allowing the high-throughput screening of expression libraries and the identification of clones producing high yields of soluble recombinant proteins.

## Materials and Methods

### Strains and Growth Conditions

*Escherichia coli* NEB 10-beta (NEB, Ipswich, Massachusetts, USA) was used for cloning and plasmid amplification. *E. coli* BL21(DE3) (Merck, Darmstadt, Germany) and *V. natriegens* V_max_ Express (SGI-DNA, La Jolla, California, USA) served as expression hosts. Unless stated otherwise, *E. coli* was grown at 37°C in LB Miller broth (LB) (Carl Roth, Karlsruhe, Germany) and Terrific Broth (TB) (Carl Roth) supplemented with 4 g/L glycerol. *V. natriegens* was cultivated in LB and TB supplemented with V2 salts (204 mM NaCl, 4.2 mM KCl, 23.14 mM MgCl_2_) at 30°C. The following antibiotics were used for plasmid selection: ampicillin (100 μg/mL), kanamycin (35 μg/mL) and spectinomycin (*E. coli* 65 μg/mL, *V. natriegens* 50 μg/mL). Unless stated otherwise, the bacteria were cultivated in 300- or 500-mL shaking flasks with four baffles, filled with 30 or 50 mL medium, respectively, in an orbital shaker at 250 rpm. The medium was inoculated to an OD_600_ of 0.1 from glycerol stocks. Protein expression was induced at an OD_600_ of 1.0 by adding IPTG to a final concentration of 1 mM. Glycerol stocks were prepared by diluting an overnight culture with medium to reach an OD_600_ of 0.1 and cultivating as above until the OD_600_ reached 1.0. At this point, the cells were centrifuged (5,000 × *g*, 15 min, 4°C) and the pellet was resuspended in ice-cold medium supplemented with 15% (v/v) glycerol. The volume was adjusted to OD_600_ = 5.0 and the vials were stored at −80°C.

### Library Cloning

Basic parts > 100 bp were synthesized (BioCat, Heidelberg, Germany) and subcloned in pUC57-Kan. Small basic parts were introduced into pUC57-Kan by PCR, using the Q5 Site-Directed Mutagenesis Kit (NEB). For assembly using the MoClo process, 40 fmol of each plasmid was mixed with 10 U T4 DNA Ligase (Promega, Mannheim, Germany), 2 μL of the corresponding buffer and 10 U BsaI or BbsI (NEB). The reaction mix was adjusted to a total volume of 20 μL with double-distilled water and incubated in a PCR cycler (PEQLAB, Erlangen, Germany) at 37°C for 30 min, followed by 20 cycles at 37°C (2 min) for digestion and 16°C (5 min) for ligation. After a final restriction step at 50°C for 10 min, the enzymes were heat-inactivated at 65°C for 5 min, and 10 μL of this MoClo reaction mix was introduced into competent *E. coli* NEB 10-beta cells as described below.

### Preparation and Transformation of Chemically Competent *E. coli*

We inoculated 300 mL LB medium with 300 μL of an overnight culture of *E. coli* NEB 10-beta or *E. coli* BL21(DE3) cells grown at 37°C to OD_600_ = 0.9. The culture was transferred to sterile 50-mL centrifugation tubes and chilled on ice for 15 min. After centrifugation (5,000 × *g*, 10 min, 4°C), each pellet was resuspended in 12.5 mL 100 mM CaCl_2_. The cells from three tubes were pooled and stored on ice for 30 min before centrifugation as above. Each pellet was resuspended in 5 mL ice-cold 100 mM CaCl_2_ supplemented with 15% (v/v) glycerol. Aliquots of 250 μL competent cells were transferred to cryogenic vials, frozen on dry ice and stored at −80°C.

For transformation, 80 μL of the competent cell suspension was mixed with 10 μL MoClo reaction mix and incubated on ice for 15 min before a 42°C heat shock for 1 min, followed by incubation on ice for 1 min. The cells were then mixed with 1 mL LB and incubated in a ThermoMixer (Eppendorf, Hamburg, Germany) at 37°C shaking at 1,000 rpm for 1 h. The cells were plated on LB agar supplemented with the corresponding antibiotic, and incubated at 37°C overnight.

### Transformation of Chemically Competent *V. natriegens*

A vial containing 50 μL of chemically competent *V. natriegens* V_max_ Express cells was thawed on ice and mixed with 200–400 ng plasmid library DNA. The cells were incubated for 30 min on ice before a 42°C heat shock for 1 min, followed by incubation on ice for 2 min. The cells were then mixed with 1 mL pre-warmed LB supplemented with V2 salts. The cells were allowed to recover in a ThermoMixer at 30°C shaking at 1,000 rpm for 2 h before streaking them on pre-warmed selection plates and incubating at 30°C overnight.

### Fluorescence Microscopy

Microscope slides with cavities were filled with boiling 1% (v/v) agarose in TAE buffer and covered with a cover glass. When the agarose had cooled, the cover glass was removed and 2 μL of culture medium was added, before applying a new cover glass. Fluorescence microscopy was carried out using a Leica DMI6000 instrument (Leica Microsystems, Wetzlar, Germany) fitted with an HCX PL FLUOTAR phase contrast objective (100 x, numerical aperture 1.3), L5 filter cube (excitation filter BP 480/40 nm, dichromatic mirror 505 nm, suppression filter BP 527/30 nm), and PhotoFluor II light source (Chroma, Bellows Falls, Vermont, USA) at 470 nm. Images were captured with a DFC360FX camera (Leica Microsystems). Brightness and contrast adjustments and image cropping were carried out using Fiji software (Schindelin et al., 2012).

### Fluorescence-Activated Cell Sorting

FACS was carried out using a BD FACSCalibur device (BD Bioscience, San Jose, California, USA), including standard laser and filter equipment. Phosphate buffered saline (PBS) was used as the sheath fluid (Thermo Fisher Scientific, Waltham, Massachusetts, USA). *E. coli* and *V. natriegens* cells grown as described above were sorted by FACS 4 h post-induction. For sample preparation, a small quantity of culture was suspended in PBS in a 5-mL test tube (VWR, Radnor, Pennsylvania, USA). The cell density was adjusted with PBS to achieve 2,000 ± 200 events per s. Forward-scatter (FSC) and side scatter (SSC) characteristics were applied to distinguish cells from background noise. The threshold was set on FSC to reduce background noise. Events of interest were gated, excited at 488 nm and analyzed for their fluorescence intensity using the 530/30 nm band-pass filter (FL1-H). A gate was applied to the top 5–10% of events with the highest fluorescence intensity, resulting in the sorting of 50,000 cells in exclusion mode. Subsequently, the cell suspension was concentrated from ~150 to 1 mL using a 0.2-μm nylon filter membrane (Merck), transferred to a flask with fresh medium and incubated overnight. The following day, serial dilutions of the culture were streaked on LB agar plates. Additionally, shaking flasks with fresh medium were inoculated from the overnight cultures at an OD_600_ of 0.1, grown until the OD_600_ reached 1.0 and induced with a final concentration of 1 mM IPTG. Four hours post-induction, the fluorescence intensity of the cells was analyzed using the same settings described above.

### Selection of High Producers

Each well of a 96-deep-well plate (VWR) was filled with 500 μL TB. Single clones picked from the agar plate previously streaked with sorted cells were used to inoculate the wells, and the cultures were grown overnight at 30°C shaking at 400 rpm. The following day, a new 96-deep-well plate was filled with 460 μL TB and inoculated with 20 μL of the overnight cultures. After cultivation for 3 h as above, recombinant protein expression was induced by adding 20 μL of IPTG to each well (final concentration 1 mM). Four hours post-induction, the plates were centrifuged (5,000 × *g*, 10 min, 4°C) and the pellets were resuspended in 25 μL BugBuster Master Mix (Merck) and incubated at room temperature for 20 min. Insoluble fractions were pelleted by centrifugation (5,000 × *g*, 20 min, 4°C) and the supernatants containing the soluble fractions were diluted in PBS. For the 12 clones showing the highest fluorescence in the soluble protein fraction, the production procedure was repeated in 50 mL TB in 500-mL shake flasks. Plasmids from these cultures were isolated using the NucleoSpin Plasmid EasyPure kit (Macherey-Nagel, Düren, Germany) according to the manufacturer's instructions before sequencing (Microsynth Sequlab, Göttingen, Germany).

### Osmotic Shock Procedure

Pellets from 2-mL cultures (see section Strains and growth conditions) were resuspended in 1 mL osmotic shock buffer 1 (20 mM Tris-HCl, 0.25 mM EDTA, 200 g/L sucrose, pH 8.0) and incubated on ice for 10 min. After centrifugation (16,000 × *g*, 10 min, 4°C) the pellet was resuspended in 1 mL osmotic shock buffer 2 (20 mM Tris-HCl, 0.25 mM EDTA, pH 8.0) and incubated as above. After further centrifugation as above, the supernatant containing the periplasmic protein fraction was transferred to a fresh reaction tube and stored at 4°C. The remaining pellet was resuspended in 100 μL BugBuster Master Mix and incubated at room temperature for 20 min. The soluble and insoluble fractions were then separated by centrifugation (16,000 × *g*, 20 min, 4°C) and 90 μL of the supernatant containing the cytoplasmic protein fraction was transferred into a fresh tube, mixed with 810 μL PBS and stored at 4°C. The remaining supernatant was discarded, and the pellet containing the insoluble and membrane protein fraction was resuspended in 1 mL PBS.

### Protein Analysis

The fluorescence intensity of each 10-μL sample of soluble protein from the periplasmic/cytoplasmic fraction was determined by mixing with 90 μL PBS, transferring to the wells of a 96-well microtiter plate and measuring the fluorescence using a Synergy HT plate reader (BioTek Instruments, Winooski, Vermont, USA) against GFP standards (Abnova, Taipei City, Taiwan), which were used to generate a standard curve. For SDS-PAGE and western blot analysis, 5–10 μL of each sample (soluble or insoluble protein in PBS) was mixed with 3.75 μL of Laemmli buffer supplemented with β-mercaptoethanol and brought to a total volume of 15 μL with double-distilled water. The sample was denatured by incubation at 95°C for 5 min and 10 μL was loaded on a 4–20% Citereon TGX Stain-Free Precast Gel (Bio-Rad, Hercules, California, USA). After running for 25 min at 250 V, gel images were captured using the ChemiDoc Imaging System (Bio-Rad). Western blots were performed using the Trans-Blot Turbo Transfer Pack and Transfer System (Bio-Rad) according to the manufacturer's instructions. Membranes were blocked with 5% bovine serum albumin (BSA) for 1 h and washed three times (5 min each) with 0.1% Tween-20 in PBS before incubation at room temperature for 2 h with the primary rat-anti-GFP 3H9 antibody (ChromoTek, Planegg-Martinsried, Germany) diluted 1:5,000 in PBS containing 0.05% Tween-20. After washing as above, the membranes were incubated at room temperature for 2 h with the horseradish peroxidase (HRP)-conjugated secondary goat anti-rat IgG antibody (Jackson ImmunoResearch Europe, Ely, UK) and Precision Protein StrepTactin-HRP Conjugate (Bio-Rad), each diluted 1:10,000. HRP-mediated luminescence was visualized using Clarity Western ECL Substrate (Bio-Rad) according to the manufacturer's protocol. Uricase activity was determined in the soluble fractions using the Amplex Red Uricase Assay Kit (Thermo Fisher Scientific) according to the manufacturer's instructions.

## Results and Discussion

### Expression Library Cloning Strategy

The combinatorial expression libraries were assembled in two consecutive cloning steps, starting from basic parts (the ribosomal binding sites, secretion tag sequences, and target protein sequences). Each of the basic parts was flanked by BsaI sites and housed in the cloning vector pUC57-Kan. Because the 4-bp BsaI overhang ends would result in frame shifts, two additional bases were introduced between the 5′ overhang and the coding sequences. For each of the five secretion tags, a synthetic ribosomal binding site was designed using the RBS Calculator (Salis et al., 2009; Espah Borujeni et al., 2014). Based on the promoter sequence and the coding sequence of the secretion tag, the ribosomal binding site was generated with the restriction of base numbers applying only to (N)_25_CAGG or (N)_30_CAGG sequences, where CAGG represents the BsaI overhang. The sites with the highest predicted translation initiation rates (TIRs) were chosen for the library (Table S1). Given that translational initiation depends not only on the ribosomal binding site but also on the subsequent coding sequence, the five ribosomal binding sites would lead to five different expression levels for each secretion tag. The TIR was predicted using a reverse calculation for each ribosomal binding site + secretion tag combination (Table 1).

**Table 1:** Organisms and expression construct combinations found in the selected clones.

**POI**	**Organism**	**RBS**	**Secretion signal**	**Fusion tag**	**Predicted T.I.R**.	**Frequency**
**IMPI**	***E. coli***	**RBS3**	**ssDmsA**	**SUMO**	**84.672**	**13/18**
	*E. coli*	RBS5	ssDmsA	MBP	630.167	4/18
	*E. coli*	RBS5	ssYahJ	GST	105.563	1/18
	*V. natriegens*	RBS2	ssYahJ	MBP	58.806	21/21
**Lucimycin**	***V. natriegens***	**RBS2**	**ssYahJ**	**His-Trx**	**58.806**	**13/21**
	*V. natriegens*	RBS2	ssDmsA	MBP	156.860	8/21
**Uricase**	***V. natriegens***	**RBS5**	**ssYahJ**	**His-Trx**	**105.563**	**10/10**
	*E. coli*	RBS2	ssYahJ	Trx	58.806	6/11
	*E. coli*	RBS4	ssTorA	GST	92.231	4/11
	*E. coli*	RBS3	ssYahJ	His-MBP	250.485	1/11

*The frequency indicates the number of clones carrying a specific combination. High producers highlighted in bold were identified as most productive clones and used for the production of the model proteins. The ribosome binding sites and secretion signal sequences are provided in the Supplementary Material*.

**Figure 1:**
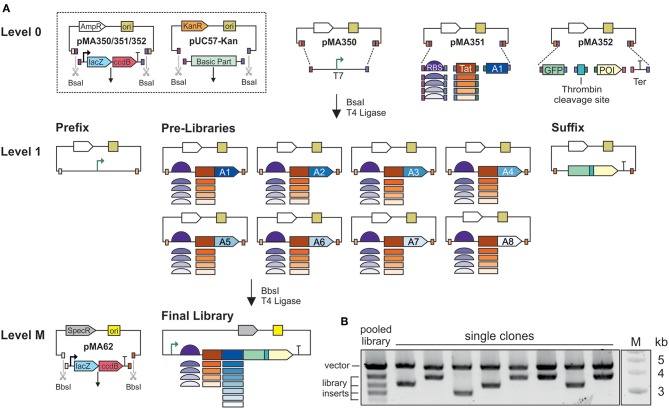
Assembly of combinatorial expression libraries. **(A)** Schematic illustration of the MoClo assembly. Basic parts are housed in pUC57-Kan, flanked by BsaI-sites. The T7 promoter is housed in pMA350. The thrombin cleavage site, green fluorescent protein (GFP), one of the proteins of interest (POI), and the terminator (Ter) are assembled in pMA352. The five ribosome binding sites (RBS) and five secretion signals are pooled, and assembled with one of the eight affinity tags (A1–A8) as Pre-libraries in pMA351. The eight affinity tags are: A1, SUMO; A2, His_6_-SUMO; A3, MBP; A4, His_6_-MBP; A5, GST; A6, His_6_-GST; A7, Trx; A8, His_6_-Trx. In Level 1, plasmids and inserts are flanked by BbsI sites. For final library assembly, the Pre-libraries are pooled and assembled with the Prefix and Suffix. **(B)** BsaI digestion of plasmids from a pooled library, and plasmids from single clones. The upper band represents the vector backbone, and the lower bands represent the assembled expression cassettes. Inserts vary in size due to the differing sizes of the affinity tags.

The first cloning step was the assembly of basic parts in Level 1 MoClo vectors (Figure 1A). We used the MoClo vectors from the Waldminhaus laboratory, which carry the *ccdB* gene in addition to *lacZ*α (Schindler et al., 2016). The *ccdB* gene encodes an inhibitor of DNA gyrase and thus kills *E. coli* cells taking up undigested or re-ligated plasmids (Bernard, 1996). This eliminates the background of undigested plasmids and increases the percentage of positive clones. Basic parts were assembled in three Level 1 vectors by Golden Gate cloning using BsaI and T4 ligase (Engler et al., 2009). The IPTG-inducible T7 promoter part, including the *lacI* repressor gene, was cloned in the first vector, pMA350, and is hereafter called *Prefix*. Pre-libraries were assembled in the second vector, pMA351. In this step, the coding sequence for one of the eight affinity tags was mixed with the five ribosomal binding sites and five secretion tags, resulting in 25 combinations. Eight Pre-libraries were prepared, one each for the eight affinity tags in the final library design. In the third vector, pMA352, we assembled the coding sequences for GFP, the thrombin cleavage site, and the protein of interest, as well as a transcriptional terminator. This building block is hereafter called *Suffix* and three versions were assembled, one for each target protein. Individual Prefix and Suffix clones were picked and verified by Sanger sequencing. Furthermore, all clones in each Pre-library (*n* = 110–900) were pooled and analyzed by Sanger sequencing, identifying the overlapping signals of the variable library elements.

The final libraries were assembled in vector pMA62, carrying a spectinomycin resistance gene and the IncP replication system (Werner et al., 2012; Schindler et al., 2016). Preliminary experiments revealed that this vector achieves efficient transformation and protein expression in *V. natriegens*. Equimolar amounts of all Pre-libraries were mixed in one tube with the Prefix, one of the three Suffix clones and the end-linker pMA671 for Golden Gate assembly using BbsI and T4 ligase. End-linkers with suitable overhangs serve as adapters whenever fewer than seven inserts are assembled in a MoClo reaction. Final libraries for each target protein were generated individually, and theoretically contained 200 combinations of ribosomal binding sites, secretion signals and affinity tags. For each library, 2,000–4,000 clones were pooled. Given a cloning efficiency of 80–100% at each step, there was a >99% probability that the final libraries covered all possible combinations. BsaI restriction analysis of the pooled libraries resulted in a pattern of five bands (Figure 1B). Given that all the ribosomal binding sites and secretion tags were similar in size, whereas the affinity tag sequences ranged from ~300 to 1,100 bp, each of the four lower bands represent the combinations with one of the affinity tags. The upper band represents the vector backbone. BsaI test digestions of plasmids from individual clones revealed the upper band but only one the four possible lower bands.

The two-stage MoClo assembly (Figure 1A) was preferred to standard Golden Gate assembly, where all basic parts are assembled in a single reaction, because as the number of parts increases the efficiency of Golden Gate assembly declines and more errors are introduced (Potapov et al., 2018). When building the libraries, none of the assembly steps involved more than four parts, and accordingly we generated large numbers of colonies at low error rates. During Level 1 and Level M cloning, sequencing revealed 87, 5% of randomly picked clones to be positive (42/48). Furthermore, when multiple parts differing in size are used to generate combinatorial libraries, there is a bias toward smaller parts. By assembling the Prefix, Pre-libraries and Suffix clones, the size differences among the basic parts were minimized. In the final libraries, we found no indication that inserts from smaller Pre-libraries were overrepresented in a manner that would lead to biased screening. Furthermore, the preparation of separate Prefix, Pre-libraries and Suffix clones makes the selection of false positives during FACS less likely. In Golden Gate cloning, single parts might not be assembled, resulting in an incomplete construct, but only whole parts would be lost. During final assembly, a small but significant number of incomplete constructs will be generated. However, the incomplete construct would either lack the promoter (missing Prefix), the ribosomal binding site and start codon (missing Pre-library) or the GFP (missing Suffix). Due to the design of the library assembly method, none of these incomplete constructs would produce a strong GFP signal. Thus, clones carrying misassembled plasmids would be excluded by FACS. For future screening with other products, only the Suffix needs to be cloned. All remaining parts can be reused. The assembly of multiple Pre-libraries means that some variants can be left out if not required and others can be added. Altogether, the design and assembly of the libraries provides a high level of modularity and flexibility.

### The Selection of High Producers

The final libraries for each target protein were introduced into the *E. coli* BL21(DE3) and *V. natriegens* V_max_ Express production strains, and the libraries in each case were separately pooled and stored as glycerol stocks. At the beginning of the selection pipeline, shaker flasks containing growth medium were inoculated from glycerol stocks to an OD_600_ of ~0.1. Recombinant protein expression was induced by adding IPTG when the culture reached an OD_600_ of ~1.0.

**Figure 2:**
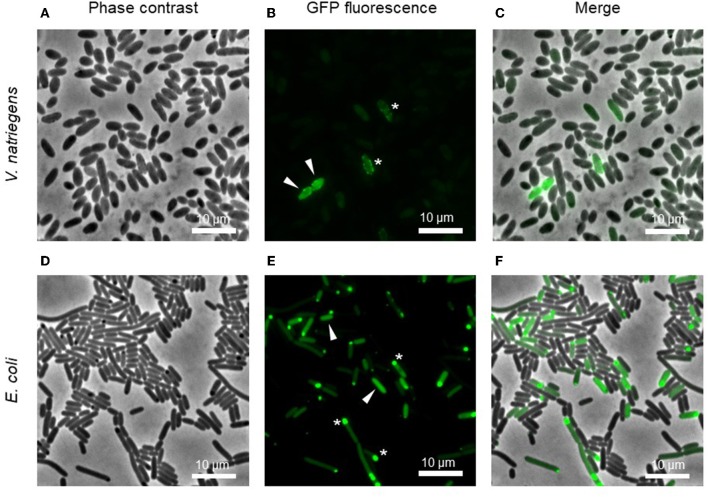
Microscopic images of pooled cultures carrying a combinatorial expression library. Arrows indicate cells with homogeneous GFP, indicating a soluble product. Stars show GFP foci, indicating insoluble inclusion bodies. Images represent the expression of the library for IMPI production in *V. natriegens* **(A–C)** and *E. coli* **(D–F)** 4 h post-induction.

Fluorescence microscopy revealed the diversity of protein expression levels in the libraries (Figure 2). We assigned the cells to three groups. The first and largest of the groups comprised cells showing no fluorescence, indicating that the fusion protein was not expressed (or expressed at levels below the detection threshold) or was expressed but inactive, perhaps due to incomplete folding. The second group comprised cells showing homogeneous fluorescence (white arrows in Figures 2B,E), indicating the expression of a soluble recombinant protein. The intensity of fluorescence varied widely in this group, including cells with very strong signals representing high producers. The final category comprised cells with punctate fluorescence (white stars in Figures 2B,E), indicating the presence of inclusion bodies.

For FACS-based selection, the cells were resuspended in PBS 4 h post-induction. Two gates were applied, the first to separate cells from background events and the second (applied to the fluorescence channel) to separate the 5–10% of cells with the highest fluorescence intensity. We sorted 50,000 cells in each of the three libraries. At a sorting rate of 30–100 events per s, these high-producer cells were separated into a large volume of PBS (100–150 mL). We therefore concentrated the suspension to 1 mL by removing the buffer using a sterile filter and a vacuum pump, and transferred the cells to a new flask for overnight incubation.

**Figure 3:**
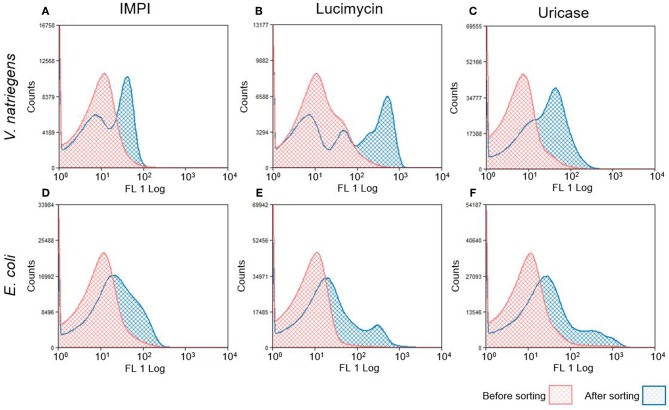
Histograms of fluorescence distribution in *V. natriegens* **(A–C)** and *E. coli* **(D–F)** cultures expressing the combinatorial libraries. Histograms in red represent GFP distribution before FACS selection. Sorted cells were grown and protein expression was induced again, as shown in blue. The shifted peak areas with regard to increased fluorescence intensity indicate a successful enrichment of induced GFP-producing cells.

The following day, the growth and induction procedures were repeated with a culture inoculated to an OD_600_ of 0.1 from the sorted overnight culture. Figure 3 shows the fluorescence intensity of cells before and after sorting. Most of the pre-sorting cells showed little or no fluorescence, although the *V. natriegens* population expressing lucimycin was a notable exception. After one round of cultivation and sorting, the fluorescence intensity of the *V. natriegens* cells had increased by approximately one order of magnitude for most of the cells producing IMPI and uricase, and by approximately two orders of magnitude for the cells producing lucimycin. The fluorescence of the pooled *E. coli* cultures also increased after sorting, although not to the extent observed with *V. natriegens*. In most cases, the fluorescence intensity remained similar to that in the original culture, although small subpopulations showed strong increases in fluorescence (Figure 3B). After two to four rounds of growth and sorting, the cells carried plasmids representing a single combination of elements from the original library. Production with this plasmid resulted in large amounts of insoluble recombinant protein (data not shown). To exploit the diversity of high producers, we therefore decided to analyze the 5–10% of cells with the highest fluorescence intensity after only one round of screening, at the cost of a higher background of less productive clones.

**Figure 4:**
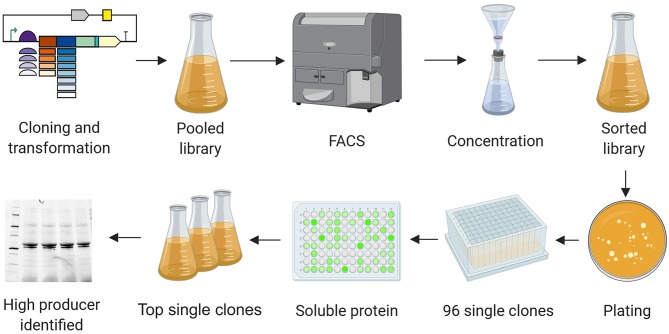
High producer selection pipeline. Combinatorial libraries were cloned and introduced into the production strains. Cultures carrying the pooled libraries were grown and expression was induced. Cells showing the highest GFP levels were selected by FACS, resulting in a cell suspension in PBS. The cell suspension was concentrated by filtration, transferred into a flask with fresh medium and grown overnight. The resulting culture was plated on agar before transferring 96 single colonies to a 96-deep-well plate. Expression was induced for 4 h. Cells were pelleted, the soluble protein fractions were extracted, and the GFP signal was quantified. The clones showing the most intense fluorescence were cultivated in flasks and subsequently analyzed by SDS-PAGE, western blot, and Sanger sequencing. Image created with BioRender.

**Figure 5:**
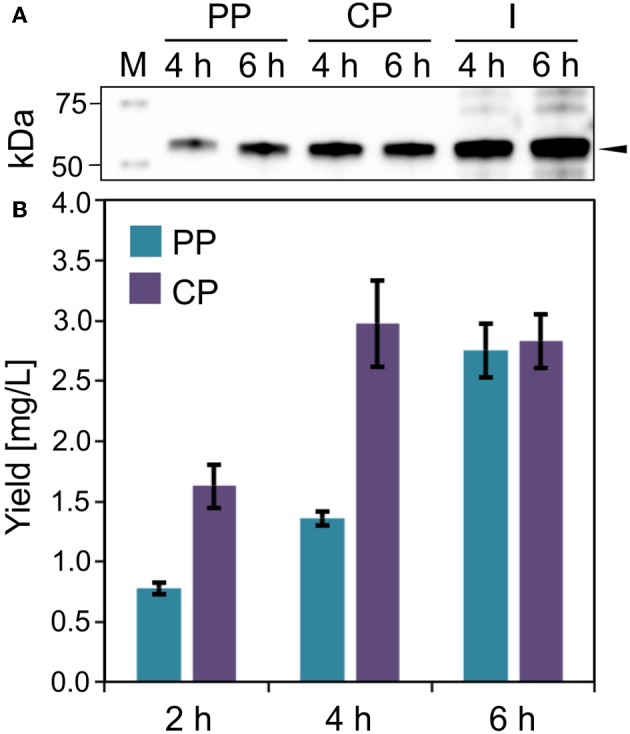
IMPI production in *E. coli*. **(A)** Western blot (anti-GFP antibody) of soluble ssDmsA-SUMO-GFP-IMPI in the periplasmic (PP), cytoplasmic (CP), and insoluble and membrane (I) fraction 4 and 6 h post-induction. The black arrow indicates the expected band at 52.8 kDa. **(B)** Fusion protein quantification in the periplasmic and cytoplasmic fractions 2, 4, and 6 h post-induction. Error bars indicate the standard deviation.

**Figure 6:**
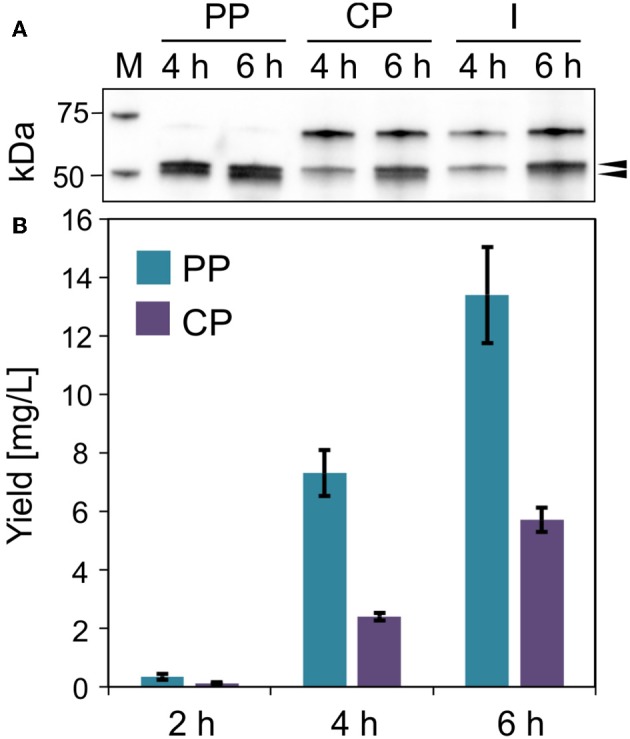
Lucimycin production in *V. natriegens*. **(A)** Western blot (anti-GFP antibody) of soluble ssYahJ-His-Trx-GFP-lucimycin in the periplasmic (PP), cytoplasmic (CP), and insoluble and membrane (I) fraction 4 and 6 h post-induction. The upper black arrow indicates the expected band at 53.2 kDa, the lower arrow indicates the putative mature protein after the cleavage of the secretion signal. **(B)** Fusion protein quantification in the periplasmic and cytoplasmic fractions 2, 4, and 6 h post-induction. Error bars indicate the standard deviation.

**Figure 7:**
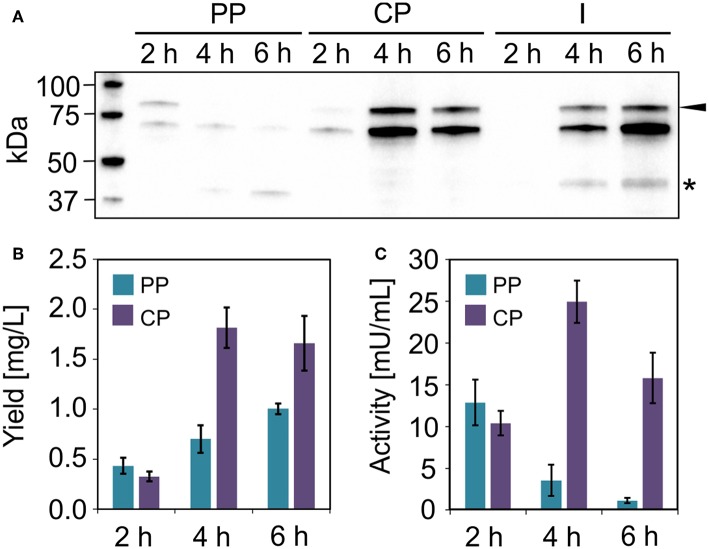
Uricase production in *V. natriegens*. **(A)** Western blot (anti-GFP antibody) of soluble ssYahJ-His-Trx-GFP-uricase in the periplasmic (PP), cytoplasmic (CP), and insoluble and membrane (I) fraction 2, 4, and 6 h post-induction. The black arrow indicates the expected band at 83.6 kDa. The star indicates a putative product resulting from incomplete translation. **(B)** Fusion protein quantification in the periplasmic and cytoplasmic fractions 2, 4, and 6 h post-induction. Error bars indicate the standard deviation. **(C)** Uricase activity in the periplasmic and cytoplasmic fractions 2, 4, and 6 h post-induction. Error bars indicate the standard deviation.

The workflow for the selection of high producers from libraries is shown in Figure 4. The day after sorting, dilutions of cells were plated on agar. Single clones were cultivated in 96-deep-well plates and protein production was induced by adding IPTG. Four hours after induction, cells were pelleted by centrifugation, and the supernatant was discarded. After lysis, the fluorescence intensity of the soluble protein fraction was measured. The top clones based on these readings were grown in shaker flasks and induced by adding IPTG. The soluble and insoluble protein fractions were analyzed by SDS-PAGE and western blot (Figures 5–7). Plasmids isolated from the top candidates were dispatched for sequencing to identify the basic elements.

### Production of Model Peptides and Proteins

The combinations of elements resulting in the highest yield for each of the three target proteins are summarized in Table 1. The highest yields of IMPI were achieved in *E. coli* using the SUMO tag. The combination ssDmsA-MBP was present in many clones at the end of the selection pipeline but this resulted in lower yields than the SUMO fusion. Interestingly, the best combination for both lucimycin and uricase was ssYahJ-His_6_-Trx in *V. natriegens*. For uricase, all clones reaching the final selection step contained this combination. For lucimycin, the combination ssDmsA-MBP was again present in many clones at the end of the selection pipeline but it achieved lower yields than ssYahJ-His_6_-Trx. Only insoluble product was observed for the production of IMPI in *V. natriegens* and uricase in *E. coli*. The high-producer expression systems highlighted in Table 1 were therefore used for the production of the three model proteins.

#### Production of IMPI

IMPI was produced in *E. coli* as a fusion to the SUMO tag and the product was exported to the periplasm mediated by the ssDmsA secretion signal. The soluble protein fractions from the periplasm and cytoplasm were separated by osmotic shock (Figure 5A). Most of the protein was located in the cytoplasm 2 and 4 h post-induction (Figure 5B). The protein yield in the cytoplasm did not increase further from 4 to 6 h post-induction. In contrast, the amount of protein in the periplasm doubled in this time. Six hours post-induction, about half of the soluble recombinant protein was located in the periplasm. At all times, a substantial quantity of insoluble product was detected (Figure 5A). IMPI contains five disulfide bonds, so translocation is likely to be accomplished by the Sec pathway, whereas the IMPI located in the cytoplasm is probably in an unfolded or incompletely folded state. Large amounts of insoluble protein result in the formation of inclusion bodies, as shown in Figure 2. The relatively low yield of 5–6 mg/L total soluble product must be regarded in the context of cell growth. Six hours post-induction, the cultures had reached an OD_600_ of only 7.5–8.0 but were still in the exponential growth phase. Given the large quantity of insoluble product, the yield of this expression system could be increased substantially by optimizing the cultivation and production process.

#### Production of Lucimycin

The antifungal peptide lucimycin was produced in *V. natriegens* with a thioredoxin tag to improve solubility and a His_6_ tag for purification by IMAC. The combination of the ssYahJ secretion signal and the selected ribosome binding site resulted in a low TIR compared to other combinations, but nevertheless achieved the highest yields (Figure 6A). Six hours post-induction the concentration of the soluble product was ~20 mg/L in total, about two thirds of which was found in the periplasmic fraction following the osmotic shock (Figure 6B). In the periplasmic fraction, a second band is visible slightly below the expected band at 53.2 kDa in the western blot (Figure 6A). The Sec and Tat translocation machineries cleave N-terminal secretion signals at the C-terminal Ala-x-Ala motif (Freudl, 2018). The removal of the secretion signal could explain the presence of the lower band. The product yield increased continually during production, and the quantity of insoluble was moderate. As for the production of IMPI in *E. coli*, the optimization of the production process could significantly increase the yields of lucimycin.

#### Production of Uricase

We were unable to identify any *E. coli* clones that achieved the production of substantial quantities of the model enzyme uricase as a soluble protein. However, moderate yields were observed when the enzyme was expressed in *V. natriegens* using the same ssYahJ-His_6_-Trx combination that achieved the highest yields of lucimycin, albeit with a different ribosome binding site and thus a higher TIR. Even so, the production and localization of the enzyme differed from lucimycin (Figure 7). The uricase fusion protein accumulated mostly in the cytoplasmic fraction, but only 2 h post-induction substantial uricase activity was also detected in the periplasmic fraction (Figure 7C). The highest yield and activity were detected 4 h post-induction, and the amount of insoluble product increased over time (Figures 7A,B). Two unexpected bands were observed in the western blot after 4 and 6 h. The dominant band slightly below the 75 kDa marker is likely to reflect non-specific antibody binding. Similar patterns were detected in all *V. natriegens* samples (see Figure 6A). The lower band between the 37 and 50 kDa markers may reflect the incomplete translation of the fusion protein, resulting in a truncated product, including GFP and N-terminal fusion tags, but missing the majority of the uricase. This would explain the increasing yield of uricase in the periplasmic fraction over time even though the activity in this fraction decreases. Translation is often interrupted by the presence of rare codons. The sequences were originally codon optimized for *E. coli*, and further analysis revealed some codons that are rarely used in *V. natriegens*. The codon CCC for proline is one example (Lee et al., 2016), and it is found at the beginning of the uricase fusion protein sequence. Replacing rare codons to match *V. natriegens* codon preferences would therefore be a starting point for the improvement of uricase production.

## Conclusions

One of the major challenges during expression screening is the formation of insoluble inclusion bodies. These often contain large quantities of active protein, leading to strong GFP signals that cannot be distinguished from the homogeneous fluorescence of soluble proteins during FACS. This issue could be addressed by including a biosensor for inclusion body formation. The transcriptomic response to inclusion body formation has been investigated, and offers multiple starting points (Baig et al., 2014). The expression of a second fluorescent marker in the presence of inclusion bodies would allow such cells to be excluded, resulting in the specific gating of cells expressing soluble recombinant proteins.

We confirmed that *V. natriegens* is a suitable host for recombinant protein production, especially in the case of uricase. Although, uricases from other species have been produced successfully in *E. coli* (Nakagawa et al., 1995; Shaaban et al., 2015), this is not the case for *L. sericata* uricase and we were similarly unable to identify any *E. coli* clones that produced this enzyme in significant amounts. In contrast, *V. natriegens* produced large amounts of the enzyme in a soluble form.

We found that many of the functional elements in *E. coli* expression constructs could also be used in *V. natriegens*, including the ssYahJ secretion signal. Most of the lucimycin product was detected in the *V. natriegens* periplasmic fraction, and the presence of an additional smaller product indicated the cleavage of the secretion signal (Figure 6A). In *E. coli*, proteins fused to thioredoxin can be released from the cytoplasm by osmotic shock (Ajouz et al., 1998), but it is unclear whether this also occurs in *V. natriegens*. We expected to detect most of the Trx-uricase fusion protein in the periplasm (Figure 7) so it is unclear why the same fusion protein combination leads to the periplasmic localization of lucimycin. The abundance of insoluble uricase indicates incomplete or incorrect folding of the large fusion protein. Given that only folded proteins are translocated by the Tat pathway, an incompletely folded protein would be expected to remain in the cytoplasm. Together with the finding that ssYahJ was the major secretion tag selected in *V. natriegens* and other high producers (data not shown), we propose that ssYahJ is recognized as a secretion signal in *V. natriegens*. To validate these results, the ssYahJ tag needs to be systematically investigated in *V. natriegens* in the absence of thioredoxin.

Taken together, the screening resulted in the identification of high producers for three difficult-to-express products in *E. coli* and *V. natriegens*. These will serve as a starting point to improve yields by optimizing the production process, including medium development and scaled-up fermentation in bioreactors. We found that the MoClo system allowed the efficient construction of combinatorial libraries containing both regulatory and coding elements. The high modularity of this screening platform will facilitate the identification of additional elements and proteins of interest, and can serve as a blueprint for novel combinatorial library screening methods in microbial and eukaryotic expression systems.

## Data Availability Statement

All datasets generated for this study are included in the manuscript/Supplementary Files.

## Author Contributions

JE conceived, designed and performed all experiments, wrote the manuscript, and coordinated its preparation. MO established the FACS and cell concentration methods, and helped during all sorting experiments. DG and TW helped to draft and revise the manuscript. PC helped to draft and revise the manuscript, and supervised the research. All authors have given their approval for this final version of the manuscript.

### Conflict of Interest

The authors declare that the research was conducted in the absence of any commercial or financial relationships that could be construed as a potential conflict of interest.

## References

[B1] Abdulrahman W. Uhring M. Kolb-Cheynel I. Garnier J.-M. Moras D. Rochel N.. (2009). A set of baculovirus transfer vectors for screening of affinity tags and parallel expression strategies. Anal. Biochem. 385, 383–385. 10.1016/j.ab.2008.10.044 19061853

[B2] Ajouz B. Berrier C. Garrigues A. Besnard M. Ghazi A. (1998). Release of thioredoxin via the mechanosensitive channel MscL during osmotic downshock of *Escherichia coli* cells. J. Biol. Chem. 273, 26670–26674. 10.1074/jbc.273.41.26670 9756908

[B3] Baig F. Fernando L. P. Salazar M. A. Powell R. R. Bruce T. F. Harcum S. W. (2014). Dynamic transcriptional response of *Escherichia coli* to inclusion body formation. Biotechnol. Bioeng. 111, 980–999. 10.1002/bit.25169 24338599 PMC3969792

[B4] Balasundaram B. Harrison S. Bracewell D. G. (2009). Advances in product release strategies and impact on bioprocess design. Trends Biotechnol. 27, 477–485. 10.1016/j.tibtech.2009.04.004 19573944

[B5] Baumann A. Skaljac M. Lehmann R. Vilcinskas A. Franta Z. (2017). Urate Oxidase produced by *Lucilia sericata* medical maggots is localized in Malpighian tubes and facilitates allantoin production. Insect Biochem. Mol. Biol. 83, 44–53. 10.1016/j.ibmb.2017.02.007 28235562

[B6] Becker W. Wimberger F. Zangger K. (2019). *Vibrio natriegens*: an alternative expression system for the high-yield production of isotopically labeled proteins. Biochemistry 58, 2799–2803. 10.1021/acs.biochem.9b00403 31199119

[B7] Berks B. C. Sargent F. Palmer T. (2000). The Tat protein export pathway. Mol. Microbiol. 35, 260–274. 10.1046/j.1365-2958.2000.01719.x 10652088

[B8] Bernard P. (1996). Positive selection of recombinant DNA by CcdB. Biotechniques 21, 320–323. 10.2144/96212pf01 8862819

[B9] Browning D. F. Richards K. L. Peswani A. R. Roobol J. Busby S. J. W. Robinson C. (2017). *Escherichia coli* “TatExpress” strains super-secrete human growth hormone into the bacterial periplasm by the Tat pathway. Biotechnol. Bioeng. 114, 2828–2836. 10.1002/bit.26434 28842980 PMC5698719

[B10] Butt T. R. Edavettal S. C. Hall J. P. Mattern M. R. (2005). SUMO fusion technology for difficult-to-express proteins. Protein Expr. Purif. 43, 1–9. 10.1016/j.pep.2005.03.016 16084395 PMC7129290

[B11] Carrió M. M. Villaverde A. (2002). Construction and deconstruction of bacterial inclusion bodies. J. Biotechnol. 96, 3–12. 10.1016/S0168-1656(02)00032-9 12142138

[B12] Choi J. H. Lee S. Y. (2004). Secretory and extracellular production of recombinant proteins using *Escherichia coli*. Appl. Microbiol. Biotechnol. 64, 625–635. 10.1007/s00253-004-1559-9 14966662

[B13] Coussement P. Maertens J. Beauprez J. van Bellegem W. de Mey M. (2014). One step DNA assembly for combinatorial metabolic engineering. Metab. Eng. 23, 70–77. 10.1016/j.ymben.2014.02.012 24594279

[B14] Dreyfus M. (1988). What constitutes the signal for the initiation of protein synthesis on *Escherichia coli* mRNAs? J. Mol. Biol. 204, 79–94. 10.1016/0022-2836(88)90601-8 2464068

[B15] Engler C. Gruetzner R. Kandzia R. Marillonnet S. (2009). Golden gate shuffling: a one-pot DNA shuffling method based on type IIs restriction enzymes. PLoS ONE 4:e5553. 10.1371/journal.pone.0005553 19436741 PMC2677662

[B16] Espah Borujeni A. Channarasappa A. S. Salis H. M. (2014). Translation rate is controlled by coupled trade-offs between site accessibility, selective RNA unfolding and sliding at upstream standby sites. Nucleic Acids Res. 42, 2646–2659. 10.1093/nar/gkt1139 24234441 PMC3936740

[B17] Espah Borujeni A. Salis H. M. (2016). Translation initiation is controlled by RNA folding kinetics via a ribosome drafting mechanism. J. Am. Chem. Soc. 138, 7016–7023. 10.1021/jacs.6b01453 27199273

[B18] Fisher A. C. Kim J.-Y. Perez-Rodriguez R. Tullman-Ercek D. Fish W. R. Henderson L. A.. (2008). Exploration of twin-arginine translocation for expression and purification of correctly folded proteins in *Escherichia coli*. Microb. Biotechnol. 1, 403–415. 10.1111/j.1751-7915.2008.00041.x 21261860 PMC3057487

[B19] Freudl R. (2018). Signal peptides for recombinant protein secretion in bacterial expression systems. Microb. Cell Fact. 17:52. 10.1186/s12934-018-0901-3 29598818 PMC5875014

[B20] Gold L. (1988). Posttranscriptional regulatory mechanisms in *Escherichia coli*. Annu. Rev. Biochem. 57, 199–233. 10.1146/annurev.bi.57.070188.001215 3052271

[B21] Gray G. L. Baldridge J. S. McKeown K. S. Heyneker H. L. Chang C. N. (1985). Periplasmic production of correctly processed human growth hormone in *Escherichia coli*: natural and bacterial signal sequences are interchangeable. Gene 39, 247–254. 10.1016/0378-1119(85)90319-1 3912261

[B22] Han Y. Guo W. Su B. Guo Y. Wang J. Chu B.. (2018). High-level expression of soluble recombinant proteins in *Escherichia coli* using an HE-maltotriose-binding protein fusion tag. Protein Expr. Purif. 142, 25–31. 10.1016/j.pep.2017.09.013 28963004

[B23] Hoffart E. Grenz S. Lange J. Nitschel R. Müller F. Schwentner A.. (2017). High substrate uptake rates empower *Vibrio natriegens* as production host for industrial biotechnology. Appl. Environ. Microbiol. 83:e01614–17. 10.1128/AEM.01614-17 28887417 PMC5666143

[B24] Hoffmann D. Ebrahimi M. Gerlach D. Salzig D. Czermak P. (2018). Reassessment of inclusion body-based production as a versatile opportunity for difficult-to-express recombinant proteins. Crit. Rev. Biotechnol. 38, 729–744. 10.1080/07388551.2017.1398134 29124949

[B25] Hoffmann D. Eckhardt D. Gerlach D. Vilcinskas A. Czermak P. (2019). Downstream processing of Cry4AaCter-induced inclusion bodies containing insect-derived antimicrobial peptides produced in *Escherichia coli*. Protein Expr. Purif. 155, 120–129. 10.1016/j.pep.2018.12.002 30529536

[B26] Joachim M. Maguire N. Schäfer J. Gerlach D. Czermak P. (2019). Process intensification for an insect antimicrobial peptide elastin-like polypeptide fusion produced in redox-engineered *Escherichia coli*. Front. Bioeng. Biotechnol. 7:150. 10.3389/fbioe.2019.00150 31316976 PMC6610315

[B27] Lee H. H. Ostrov N. Wong B. G. Gold M. A. Khalil A. Church G. M. (2016). *Vibrio natriegens*, a new genomic powerhouse. bioRxiv 058487. 10.1101/058487

[B28] Loughran S. T. Bree R. T. Walls D. (2017). Purification of polyhistidine-tagged proteins. Methods Mol. Biol. 1485, 275–303. 10.1007/978-1-4939-6412-3_14 27730558

[B29] Mahr R. Boeselager R. F. von Wiechert J. Frunzke J. (2016). Screening of an *Escherichia coli* promoter library for a phenylalanine biosensor. Appl. Microbiol. Biotechnol. 100, 6739–6753. 10.1007/s00253-016-7575-8 27170323

[B30] Mambetisaeva E. T. Martin P. E. Evans W. H. (1997). Expression of three functional domains of connexin 32 as thioredoxin fusion proteins in *Escherichia coli* and generation of antibodies. Protein Expr. Purif. 11, 26–34. 10.1006/prep.1997.0761 9325135

[B31] McNiff M. L. Haynes E. P. Dixit N. Gao F. P. Laurence J. S. (2016). Thioredoxin fusion construct enables high-yield production of soluble, active matrix metalloproteinase-8 (MMP-8) in *Escherichia coli*. Protein Expr. Purif. 122, 64–71. 10.1016/j.pep.2016.02.012 26923061 PMC5137367

[B32] Moore S. J. Lai H.-E. Kelwick R. J. R. Chee S. M. Bell D. J. Polizzi K. M.. (2016). EcoFlex: a multifunctional MoClo kit for *E. coli* synthetic biology. ACS Synth. Biol. 5, 1059–1069. 10.1021/acssynbio.6b00031 27096716

[B33] Nakagawa S. Oda H. Anazawa H. (1995). High cell density cultivation and high recombinant protein production of *Escherichia coli* strain expressing uricase. Biosci. Biotechnol. Biochem. 59, 2263–2267. 10.1271/bbb.59.2263 8611749

[B34] Oliveira J. E. de Soares C. R. Peroni C. N. Gimbo E. Camargo I. M. Morganti L.. (1999). High-yield purification of biosynthetic human growth hormone secreted in *Escherichia coli* periplasmic space. J. Chromatogr. A 852, 441–450. 10.1016/S0021-9673(99)00613-5 10481982

[B35] Pöppel A.-K. Koch A. Kogel K.-H. Vogel H. Kollewe C. Wiesner J.. (2014). Lucimycin, an antifungal peptide from the therapeutic maggot of the common green bottle fly *Lucilia sericata*. Biol. Chem. 395, 649–656. 10.1515/hsz-2013-0263 24622788

[B36] Potapov V. Ong J. L. Kucera R. B. Langhorst B. W. Bilotti K. Pryor J. M.. (2018). Comprehensive profiling of four base overhang ligation fidelity by T4 DNA ligase and application to DNA assembly. ACS Synth. Biol. 7, 2665–2674. 10.1021/acssynbio.8b00333 30335370

[B37] Punginelli C. Ize B. Stanley N. R. Stewart V. Sawers G. Berks B. C.. (2004). mRNA secondary structure modulates translation of Tat-dependent formate dehydrogenase N. J. Bacteriol. 186, 6311–6315. 10.1128/JB.186.18.6311-6315.2004 15342602 PMC515163

[B38] Ramazzina I. Folli C. Secchi A. Berni R. Percudani R. (2006). Completing the uric acid degradation pathway through phylogenetic comparison of whole genomes. Nat. Chem. Biol. 2, 144–148. 10.1038/nchembio768 16462750

[B39] Reuten R. Nikodemus D. Oliveira M. B. Patel T. R. Brachvogel B. Breloy I.. (2016). Maltose-binding protein (MBP), a secretion-enhancing tag for mammalian protein expression systems. PLoS ONE 11:e0152386. 10.1371/journal.pone.0152386 27029048 PMC4814134

[B40] Saïda F. (2007). Overview on the expression of toxic gene products in Escherichia coli. Curr. Prot. Protein Sci. Chapter 5, Unit 5.19. 10.1002/0471140864.ps0519s50 18429327

[B41] Salis H. M. Mirsky E. A. Voigt C. A. (2009). Automated design of synthetic ribosome binding sites to control protein expression. Nat. Biotechnol. 27, 946–950. 10.1038/nbt.1568 19801975 PMC2782888

[B42] Schäfer F. Seip N. Maertens B. Block H. Kubicek J. (2015). Purification of GST-tagged proteins. Meth. Enzymol. 559, 127–139. 10.1016/bs.mie.2014.11.005 26096507

[B43] Schindelin J. Arganda-Carreras I. Frise E. Kaynig V. Longair M. Pietzsch T.. (2012). Fiji: an open-source platform for biological-image analysis. Nat. Methods 9, 676–682. 10.1038/nmeth.2019 22743772 PMC3855844

[B44] Schindler D. Milbredt S. Sperlea T. Waldminghaus T. (2016). Design and assembly of DNA sequence libraries for chromosomal insertion in bacteria based on a set of modified MoClo vectors. ACS Synth. Biol. 5, 1362–1368. 10.1021/acssynbio.6b00089 27306697

[B45] Schleicher L. Muras V. Claussen B. Pfannstiel J. Blombach B. Dibrov P.. (2018). *Vibrio natriegens* as host for expression of multisubunit membrane protein complexes. Front. Microbiol. 9:2537. 10.3389/fmicb.2018.02537 30410475 PMC6209661

[B46] Schreiber C. Müller H. Birrenbach O. Klein M. Heerd D. Weidner T.. (2017). A high-throughput expression screening platform to optimize the production of antimicrobial peptides. Microb. Cell Fact. 16:29. 10.1186/s12934-017-0637-5 28193216 PMC5307881

[B47] Shaaban M. I. Abdelmegeed E. Ali Y. M. (2015). Cloning, expression, and purification of recombinant uricase enzyme from *Pseudomonas aeruginosa* Ps43 using *Escherichia coli*. J. Microbiol. Biotechnol. 25, 887–892. 10.4014/jmb.1410.10041 25588559

[B48] Sinah N. Williams C. A. Piper R. C. Shields S. B. (2012). A set of dual promoter vectors for high throughput cloning, screening, and protein expression in eukaryotic and prokaryotic systems from a single plasmid. BMC Biotechnol. 12:54. 10.1186/1472-6750-12-54 22916790 PMC3527174

[B49] Steinmetz E. J. Auldridge M. E. (2017). Screening fusion tags for improved recombinant protein expression in *E. coli* with the Expresso® solubility and expression screening system. Curr. Prot. Protein Sci. 90, 5.27.1-5.27.20. 10.1002/cpps.39 29091274 PMC5791521

[B50] Tsirigotaki A. Geyter J. de Šoštaric N. Economou A. Karamanou S. (2017). Protein export through the bacterial Sec pathway. Nat. Rev. Microbiol. 15, 21–36. 10.1038/nrmicro.2016.161 27890920

[B51] Tullman-Ercek D. DeLisa M. P. Kawarasaki Y. Iranpour P. Ribnicky B. Palmer T.. (2007). Export pathway selectivity of *Escherichia coli* twin arginine translocation signal peptides. J. Biol. Chem. 282, 8309–8316. 10.1074/jbc.M610507200 17218314 PMC2730154

[B52] Wedde M. Weise C. Kopacek P. Franke P. Vilcinskas A. (1998). Purification and characterization of an inducible metalloprotease inhibitor from the hemolymph of greater wax moth larvae, *Galleria mellonella*. Eur. J. Biochem. 255, 535–543. 10.1046/j.1432-1327.1998.2550535.x 9738891

[B53] Weinstock M. T. Hesek E. D. Wilson C. M. Gibson D. G. (2016). *Vibrio natriegens* as a fast-growing host for molecular biology. Nat. Methods 13, 849–851. 10.1038/nmeth.3970 27571549

[B54] Werner S. Engler C. Weber E. Gruetzner R. Marillonnet S. (2012). Fast track assembly of multigene constructs using Golden Gate cloning and the MoClo system. Bioeng. Bugs 3, 38–43. 10.4161/bbug.3.1.18223 22126803

[B55] Young C. L. Britton Z. T. Robinson A. S. (2012). Recombinant protein expression and purification: a comprehensive review of affinity tags and microbial applications. Biotechnol. J. 7, 620–634. 10.1002/biot.201100155 22442034

