# Supplementary material for: Selection of High Producers From Combinatorial Libraries for the Production of Recombinant Proteins in Escherichia coli and Vibrio natriegens
Source: Front Bioeng Biotechnol. 2019 Oct 4;7:254. doi: 10.3389/fbioe.2019.00254 (PMC6788121; doi:10.3389/fbioe.2019.00254)
Supplement: Supplementary file 1 [file Table_1.pdf]

High producer selection from combinatorial libraries for recombinant protein and peptide production in *E. coli* and *V. natriegens*

Joel Eichmann[1,2], Markus Oberpaul[3], Tobias Weidner[1], Doreen Gerlach[3], Peter Czermak[1,2,3*]

[1] Institute of Bioprocess Engineering and Pharmaceutical Technology, University of Applied Sciences Mittelhessen, Giessen, Germany

[2] Faculty of Biology and Chemistry, Justus-Liebig University of Giessen, Germany

[3] Branch for Bioresources, Fraunhofer Institute for Molecular Biology and Applied Ecology, Giessen, Germany

*** Correspondence:**
Prof. Dr.-Ing. Peter Czermak
peter.czermak@lse.thm.de

Supplementary material

1 Sequences

1.1 Synthetic RBS

Supplemental Table 1: Sequences of synthetic RBS, designed using the RBS calculator (Salis et al. 2009; Espah Borujeni et al. 2014). Underlined sequences indicate BsaI-overhangs for Golden Gate cloning.

RBS	Secretion signal	RBS sequence 5‘ - 3‘
RBS1	ssNapA	GGAGAGTCTACAATATAATATAAGGAGGTCAGG
RBS2	ssTorA	GGAGTTCCATACATAAACAATAGGGAGGACACAACAGG
RBS3	ssYahJ	GGAGTAATAACCAAAACAAAAGAGGAGGTACCAACAGG
RBS4	ssYcdB	GGAGTATAATAAACAGTAGATAAGGAGGTAATAACAGG
RBS5	ssDmsA	GGAGCCTAGAGATTCTAAGATAAGGAGGTAAATACAGG

1.2 Secretion signals

Supplementals Table 2: Sequences of the secretion signals used for RBS design and periplasmic translocation. Underlined sequences indicate BsaI-overhangs for Golden Gate cloning.

Secretion signal	Peptide sequence	DNA sequence 5’ – 3’
ssNapA	MKLSRRSFMKANAVAAAAAAAGLSVPGVARAVVGQ	CAGGATGAAACTCAGTCGTCGTAGCTTTATGAAAGCTAACGCCGTTGCGGCCGCTGCGGCGGCTGCCGGTCTGAGCGTGCCGGGCGTTGCCCGCGCCGTTGTTGGTCAGAGTG
ssTorA	MNNNDLFQASRRRFLAQLGGLTVAGMLGPSLLTPRRATAAQAATDA	CAGGATGAACAATAACGATCTCTTTCAGGCATCACGTCGGCGTTTTCTGGCACAACTCGGCGGCTTAACCGTCGCCGGGATGCTGGGGCCGTCATTGTTAACGCCGCGACGTGCGACTGCGGCGCAAGCGGCGACTGACGCTAGTG
ssYahJ	MKESNSRREFLSQSGKMVTAAALFGTSVPLAHAAVAGTL	CAGGATGAAAGAAAGCAATAGCCGCCGTGAATTTCTGAGCCAGAGCGGTAAGATGGTCACCGCCGCCGCGCTGTTTGGTACCTCTGTGCCGCTCGCCCATGCGGCGGTAGCTGGCACCCTAAGTG
ssYcdB	MQYKDENGVNEPSRRRLLKVIGALALAGSCPVAHAQKTQSA	CAGGATGCAGTATAAAGATGAAAACGGCGTGAATGAACCGTCACGCCGACGTTTACTGAAAGTGATAGGTGCACTGGCGCTGGCGGGAAGTTGTCCGGTCGCTCATGCACAAAAAACGCAAAGTGCGAGTG
ssDmsA	MKTKIPDAVLAAEVSRRGLVKTTAIGGLAMASSALTLPFSRIAHA	CAGGATGAAAACCAAAATTCCGGATGCGGTGCTGGCGGCGGAAGTGTCCCGTCGTGGCCTGGTGAAAACCACCGCGATTGGCGGCCTGGCGATGGCGTCCTCCGCGCTGACCCTGCCGTTTTCCCGTATTGCGCATGCGAGTG

1.3 Plasmid sequences from identified high producers (expression cassette only)

Bold characters indicate the T7-promoter, underlined characters indicate the RBS.

> High-producer 1 (IMPI)

;T7::RBS3_ssDmsA-SUMO-GFP-Thr-IMPI_T7ter

TAATACGACTCACTATAGGGGAATTGTGAGCGGATAACAATTCCCCTCTAGAAATAAC
TATTTACGGAGTAATAACCAAAACAAAAGAGGAGGTACCAACAGGATGAAAACCAAAA
TTCCGGATGCGGTGCTGGCGGCGGAAGTGTCCCGTCGTGGCCTGGTGAAAACCACCGCG
ATTGGCGGCCTGGCGATGGCGTCCTCCGCGCTGACCCTGCCGTTTTCCCGTATTGCGCAT
GCGAGTGAAGGTTCTTCTATGGCTAGCATGTCGGACTCAGAAGTCAATCAAGAAGCTAA
GCCAGAGGTCAAGCCAGAAGTCAAGCCTGAGACTCACATCAATTTAAAGGTGTCCGATG
GATCTTCAGAGATCTTCTTCAAGATCAAAAAGACCACTCCTTTAAGAAGGCTGATGGAA
GCGTTCGCTAAAAGACAGGGTAAGGAAATGGACTCCTTAAGATTCTTGTACGACGGTAT
TAGAATTCAAGCTGATCAGACCCCTGAAGATTTGGACATGGAGGATAACGATATTATTG
AGGCTCACAGAGAACAGATTGGTGGATCGAGCTATCAGAGGAGAGCAAAGGCGAAGA
ACTGTTTACGGGCGTCGTGCCGATTCTGGTGGAACTGGATGGCGATGTCAATGGTCATA
AATTCAGCGTTAGCGGCGAAGGTGAAGGCGATGCGACCTATGGTAAACTGACGCTGAA
ATTTATTTGCACCACGGGTAAACTGCCGGTGCCGTGGCCGACCCTGGTTACCACGCTGAC
GTATGGTGTTCAGTGTTTCTCCCGCTACCCGGATCATATGAAACAACACGACTTTTTCAA
ATCAGCGATGCCGGAAGGTTATGTCCAGGAACGTACCATTTTCTTTAAAGATGACGGCA
ACTACAAAACCCGCGCCGAAGTCAAATTTGAAGGTGATACGCTGGTGAACCGTATTGAA
CTGAAAGGCATCGATTTCAAAGAAGATGGTAATATCCTGGGCCATAAACTGGAATATAA
CTACAATTCGCACAACGTTTACATTATGGCAGATAAACAGAAAAACGGTATCAAAGTCA
ACTTCAAAATCCGCCATAATATCGAAGATGGCTCTGTGCAACTGGCTGACCACTATCAG
CAAAACACCCCGATCGGTGATGGCCCGGTTCTGCTGCCGGACAATCATTACCTGAGCAC
CCAGTCTGCACTGAGTAAAGATCCGAACGAAAAACGTGACCACATGGTCCTGCTGGAAT
TTGTCACGGCGGCGGGTATCACGCTGGGTATGGATGAACTGTATAAAGAGCAAGGCGGC
GGCAGCCTGGTGCCGCGCGGAAGTATTGTGCTGATTTGTAACGGTGGTCACGAATACTA
CGAATGTGGCGGTGCTTGCGATAATGTCTGTGCTGATCTGCATATCCAAAACAAAACCA
ACTGCCCGATCATCAACATCCGTTGTAACGATAAATGCTACTGTGAAGATGGCTACGCG
CGCGACGTGAATGGTAAATGCATTCCGATCAAAGACTGTCCGAAAATCCGTAGCTAATG
GGTAATTGATTAATACCTAGGCTGCTAAACAAAGCCCGAAAGGAAGCTGAGTTGGCTGC
TGCCACCGCTGAGCAATAACTAGCATAACCCCTTGGGGCCTCTAAACGGGTCTTGAGGG
GTTTTTTG

> High-producer 2 (Lucimycin)

;T7::RBS2_ssYahJ-His-Trx-GFP-Thr-Lucimycin_T7ter

TAATACGACTCACTATAGGGGAATTGTGAGCGGATAACAATTCCCCTCTAGAAATAAC
TATTTACGGAGTTCCATACATAAACAATAGGGAGGACACAACAGGATGAAAGAAAGCA
ATAGCCGCCGTGAATTTCTGAGCCAGAGCGGTAAGATGGTCACCGCCGCCGCGCTGTTT
GGTACCTCTGTGCCGCTCGCCCATGCGGCGGTAGCTGGCACCCTAAGTGAACATCATCA
CCATCACCACAGCGACAAAATTATCCACCTGACGGACGACAGTTTCGACACGGATGTTC
TGAAAGCCGATGGCGCGATTCTGGTTGACTTCTGGGCGGAATGGTGCGGCCCGTGTAAA
ATGATTGCACCGATCCTGGATGAAATTGCTGACGAATATCAGGGCAAACTGACCGTGGC
GAAACTGAACATCGATCAAAATCCGGGTACCGCCCCGAAATACGGCATTCGTGGTATCC
CGACGCTGCTGCTGTTTAAAAACGGTGAAGTTGCGGCCACCAAAGTCGGTGCCCTGTCA
AAAGGTCAACTGAAAGAATTCCTGGACGCAAATCTGGCGCTATCAGAGGAGAGCAAAG
GCGAAGAACTGTTTACGGGCGTCGTGCCGATTCTGGTGGAACTGGATGGCGATGTCAAT
GGTCATAAATTCAGCGTTAGCGGCGAAGGTGAAGGCGATGCGACCTATGGTAAACTGAC
GCTGAAATTTATTTGCACCACGGGTAAACTGCCGGTGCCGTGGCCGACCCTGGTTACCA
CGCTGACGTATGGTGTTCAGTGTTTCTCCCGCTACCCGGATCATATGAAACAACACGACT
TTTTCAAATCAGCGATGCCGGAAGGTTATGTCCAGGAACGTACCATTTTCTTTAAAGATG
ACGGCAACTACAAAACCCGCGCCGAAGTCAAATTTGAAGGTGATACGCTGGTGAACCGT
ATTGAACTGAAAGGCATCGATTTCAAAGAAGATGGTAATATCCTGGGCCATAAACTGGA
ATATAACTACAATTCGCACAACGTTTACATTATGGCAGATAAACAGAAAAACGGTATCA
AAGTCAACTTCAAAATCCGCCATAATATCGAAGATGGCTCTGTGCAACTGGCTGACCAC
TATCAGCAAAACACCCCGATCGGTGATGGCCCGGTTCTGCTGCCGGACAATCATTACCT
GAGCACCCAGTCTGCACTGAGTAAAGATCCGAACGAAAAACGTGACCACATGGTCCTGC
TGGAATTTGTCACGGCGGCGGGTATCACGCTGGGTATGGATGAACTGTATAAAGAGCAA
GGCGGCGGCAGCCTGGTGCCGCGCGGAAGTCAGCACGGTTACGGCGCAGGTGGTCACG
GTCAGCAGGGTTACGGCTCGCAACACTCGTCGCACGCTCCGCAAGGCGGTCACGTCGTC
CGTGAACAGGGTTTTAGTGGCCACGTGCATGAACAGCAAGCGGGTCATCACCATGAAGC
CGGCCACCATGAACAGGCGGGTCACCATGAACAAAGCGGCCAGCAAGTTCACGGCCAG
GGTCATGGCTATAAATCTCACGGTTACTAATGGGTAATTGATTAATACCTAGGCTGCTAA
ACAAAGCCCGAAAGGAAGCTGAGTTGGCTGCTGCCACCGCTGAGCAATAACTAGCATA
ACCCCTTGGGGCCTCTAAACGGGTCTTGAGGGGTTTTTTG

> High-producer 3 (Uricase)

;T7::RBS5_ssYahJ-His-Trx-GFP-Thr-Uricase_T7ter

TAATACGACTCACTATAGGGGAATTGTGAGCGGATAACAATTCCCCTCTAGAAATAAC
TATTTACGGAGCCTAGAGATTCTAAGATAAGGAGGTAAATACAGGATGAAAGAAAGCA
ATAGCCGCCGTGAATTTCTGAGCCAGAGCGGTAAGATGGTCACCGCCGCCGCGCTGTTT
GGTACCTCTGTGCCGCTCGCCCATGCGGCGGTAGCTGGCACCCTAAGTGAACATCATCA
CCATCACCACAGCGACAAAATTATCCACCTGACGGACGACAGTTTCGACACGGATGTTC
TGAAAGCCGATGGCGCGATTCTGGTTGACTTCTGGGCGGAATGGTGCGGCCCGTGTAAA
ATGATTGCACCGATCCTGGATGAAATTGCTGACGAATATCAGGGCAAACTGACCGTGGC
GAAACTGAACATCGATCAAAATCCGGGTACCGCCCCGAAATACGGCATTCGTGGTATCC
CGACGCTGCTGCTGTTTAAAAACGGTGAAGTTGCGGCCACCAAAGTCGGTGCCCTGTCA
AAAGGTCAACTGAAAGAATTCCTGGACGCAAATCTGGCGCTATCAGAGGAGAGCAAAG
GCGAAGAACTGTTTACGGGCGTCGTGCCGATTCTGGTGGAACTGGATGGCGATGTCAAT
GGTCATAAATTCAGCGTTAGCGGCGAAGGTGAAGGCGATGCGACCTATGGTAAACTGAC
GCTGAAATTTATTTGCACCACGGGTAAACTGCCGGTGCCGTGGCCGACCCTGGTTACCA
CGCTGACGTATGGTGTTCAGTGTTTCTCCCGCTACCCGGATCATATGAAACAACACGACT
TTTTCAAATCAGCGATGCCGGAAGGTTATGTCCAGGAACGTACCATTTTCTTTAAAGATG
ACGGCAACTACAAAACCCGCGCCGAAGTCAAATTTGAAGGTGATACGCTGGTGAACCGT
ATTGAACTGAAAGGCATCGATTTCAAAGAAGATGGTAATATCCTGGGCCATAAACTGGA
ATATAACTACAATTCGCACAACGTTTACATTATGGCAGATAAACAGAAAAACGGTATCA
AAGTCAACTTCAAAATCCGCCATAATATCGAAGATGGCTCTGTGCAACTGGCTGACCAC
TATCAGCAAAACACCCCGATCGGTGATGGCCCGGTTCTGCTGCCGGACAATCATTACCT
GAGCACCCAGTCTGCACTGAGTAAAGATCCGAACGAAAAACGTGACCACATGGTCCTGC
TGGAATTTGTCACGGCGGCGGGTATCACGCTGGGTATGGATGAACTGTATAAAGAGCAA
GGCGGCGGCAGCCTGGTGCCGCGCGGAAGTTTTGCTCGTCCCTTGCAGCGTCCTAGTGG
AAAGGGGACACCGCAGCAAGATCGCGAAGCCCCTCATCAGTACACGATCTCCGATCATG
GGTATGGCAAAGACAGTGTTAAAGTCCTGCATGTTAAACGCGATGGCCCAGTACACTCG
ATCAAAGAGTTTGAGGTAGGCACCCATCTGAAACTCTACTCCAAGAAAGACTACTTTCA
TGGCGACAATAGCGATATCGTGGCGACTGACAGCCAGAAAAATACCGTATATCTGCTCG
CGAAGAAATTTGGCATTGAGAATCCAGAGAAATTTGGGCTTATTCTTGCATCGCACTTCC
TGAACAAATATGCGCATGTCGAAGAAGTGCACATTCACGTGGAAGAATATCCCTGGCAA
CGGATTTGCCAGGATCAAGTTGGTGGTACAACCGGCGCATGTGGTGATGCTCCGAACTT
CAGTACGTTCAATAATCGCCAGAAACACAACCATGCCTTCATTTTCACCCCTACGGAAGT
TCGCTATTGCGATGTGGTGTTACGTCGTACTGAACCGAAACAGACCGTAATTAGCGGTA
TTCGTGGACTGCGGGTTCTGAAAACGACGCAAAGTAGCTTCGTGAACTTTGTGAACGAC
GAATTTCGCTCTCTGCCGGATCAGTATGACCGCATTTTCTCAACCATCGTCGATTGCTCTT
GGGAATACTCACGCACCAATAATGTCAAATTCTGTCAGGACTGGAATACGGTGAAGAAC
ATCATCATCAAGAAATTCGCGGGTGATCCGAATGTTGGCACTTCAAGCCCGTCGGTGCA
ACATACTCTGTATCTGACCGAGAAAGAAGTCTTAGATGCCTTGCCAGAAGTATCCGTGA
TTAGCATGACCATGCCGAACAAACACTACTTTAACTTTGACACAAAACCGTTTCAGTCG
GTTGTTCCAGGTGAAAACAACGAGGTGTTTATCCCGGTCGATAAACCGCATGGCACCAT
TTACGCACAACTGGCGCGCAAGGATCTGGCCTCTCACTTATAATGGGTAATTGATTAATA
CCTAGGCTGCTAAACAAAGCCCGAAAGGAAGCTGAGTTGGCTGCTGCCACCGCTGAGCA
ATAACTAGCATAACCCCTTGGGGCCTCTAAACGGGTCTTGAGGGGTTTTTTG

2 High Producers

SDS-PAGE and / or Western Blot of top candidates shown in Table 1.

2.1 IMPI

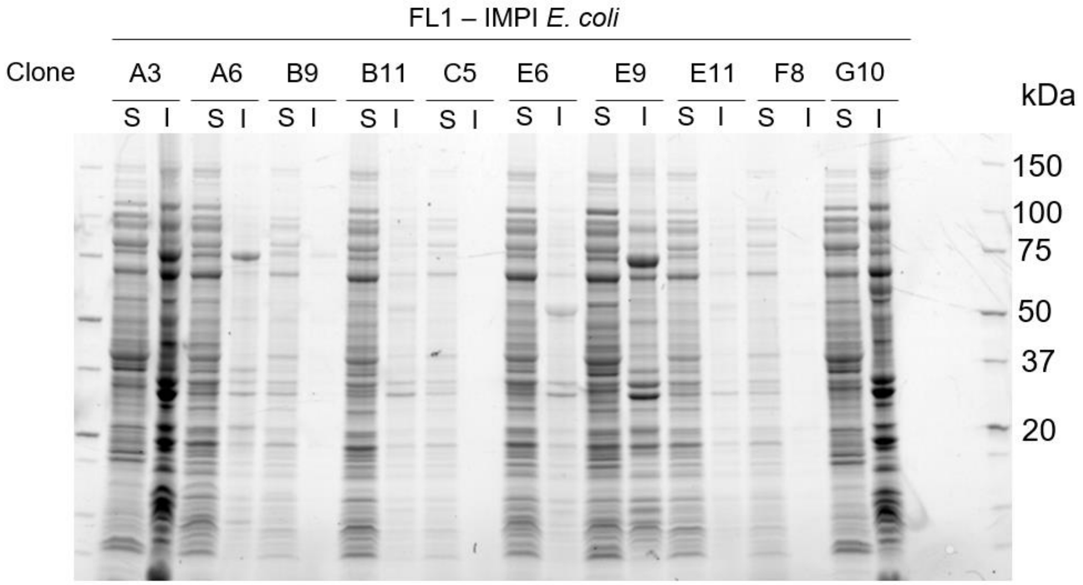

Supplementary Figure 1: SDS-PAGE of ten top candidates for IMPI production in *E. coli*. Standard: Precision Plus Protein™ Unstained Protein Standard. S: soluble protein fraction; I: insoluble protein fraction.

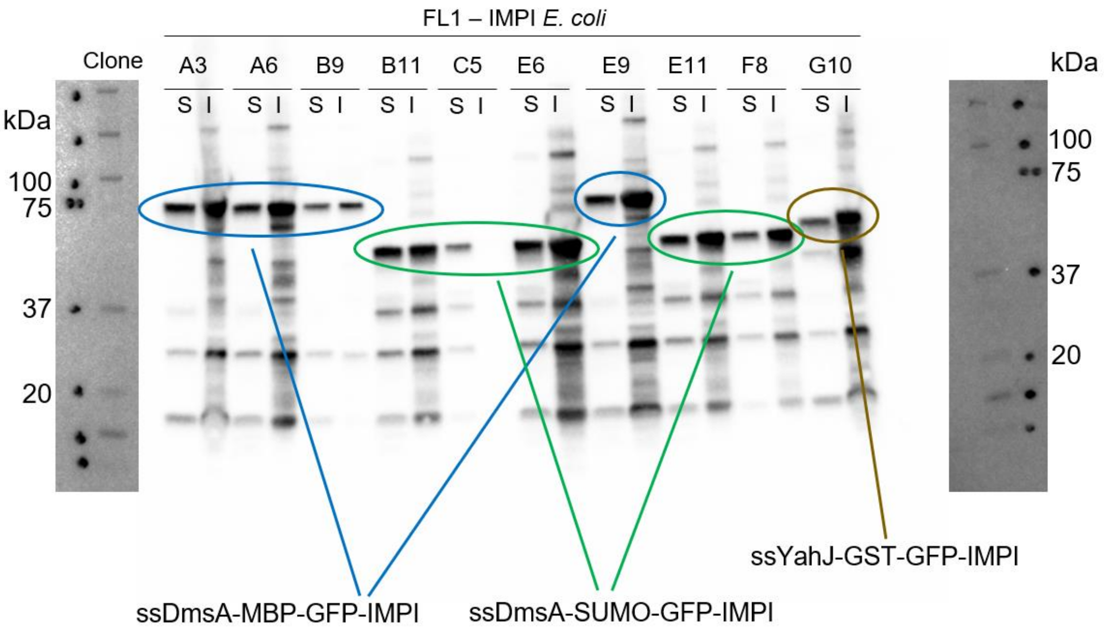

Supplementary Figure 2: Western Blot of ten top candidates for IMPI production in *E. coli*. S: soluble protein fraction; I: insoluble protein fraction. Circles indicate the expected Bands according to sequencing of the expression plasmids. Primary antibody: Anti-GFP 3H9 (Chromotek), secondary antibody: Anti-Rat-HRP (Jackson Immuno).

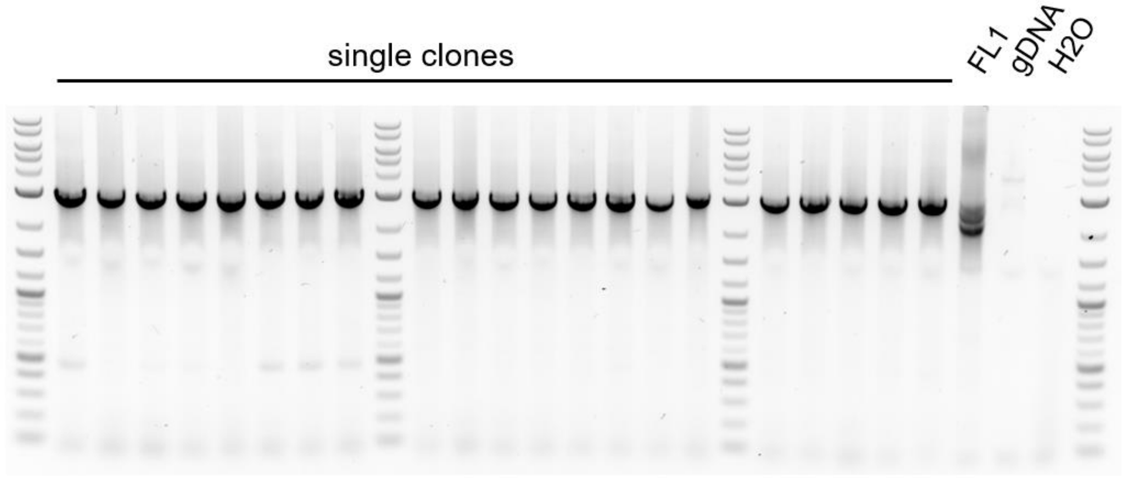

Supplementary Figure 3: Colony PCR of selected clones for IMPI production in *V. natriegens*. Controls: Combinatorial expression library FL1, genomic *V. natriegens* DNA, water. Sanger sequencing of the PCR products revealed all clones to carry the same expression plasmid: RBS2_ssYahJ-MBP-GFP-IMPI. Production resulted in insoluble product.

2.2 Lucimycin

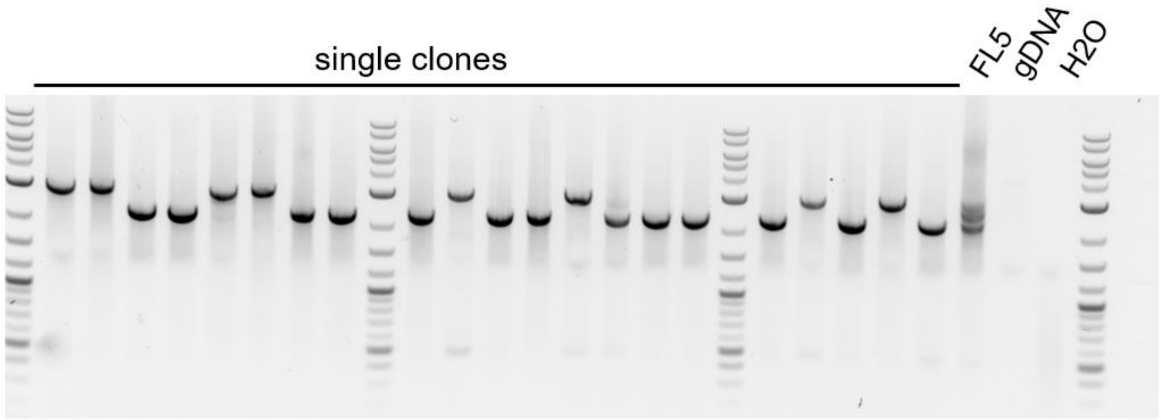

Supplementary Figure 4: Colony PCR of selected clones for Lucimycin production in *V. natriegens*. Controls: Combinatorial expression library FL5, genomic *V. natriegens* DNA, water. Sanger sequencing of the PCR products revealed two expression plasmids: RBS2_ssDmsA-MBP-GFP-Lucimycin (upper bands) and RBS2_YahJ-His$_6$-Trx-GFP-Lucimycin (lower bands).

2.3 Uricase

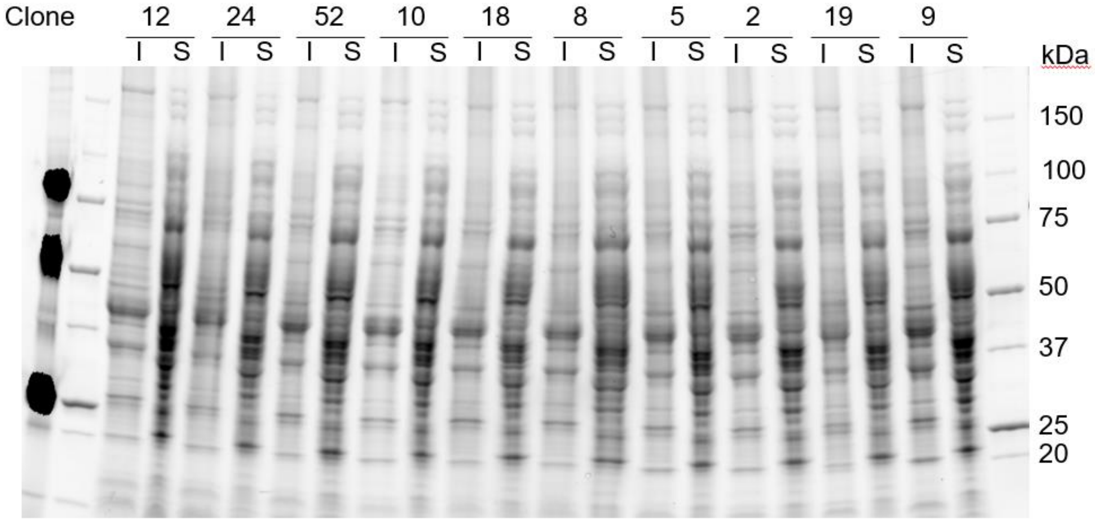

Supplementary Figure 5: SDS-PAGE of ten top candidates for Uricase production in *V. natriegens*. Standard right: Precision Plus Protein™ Unstained Protein Standard (Biorad). Standards left: Precision Plus Protein™ Western C Blotting Standard (BioRad) S: soluble protein fraction; I: insoluble protein fraction.

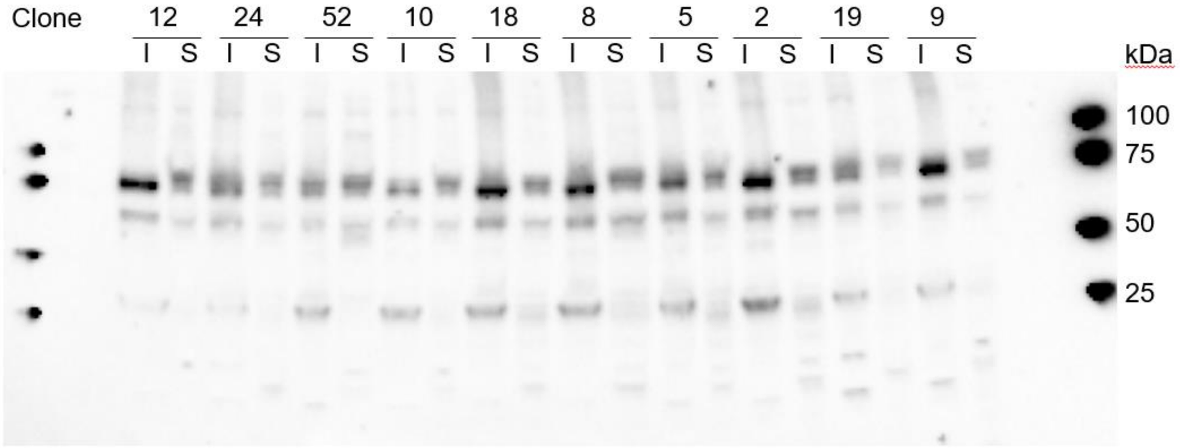

Supplementary Figure 6: Western Blot of ten top candidates for Uricase production in *V. natriegens.* Antibody: Anti-GFP-HRP GF28R (Thermo Fisher Scientific). S: soluble protein fraction; I: insoluble protein fraction.

3 Cropped images

3.1 Western Blots

Multiple cropped images of Western Blots are shown in the main article. Here, the full images are shown.

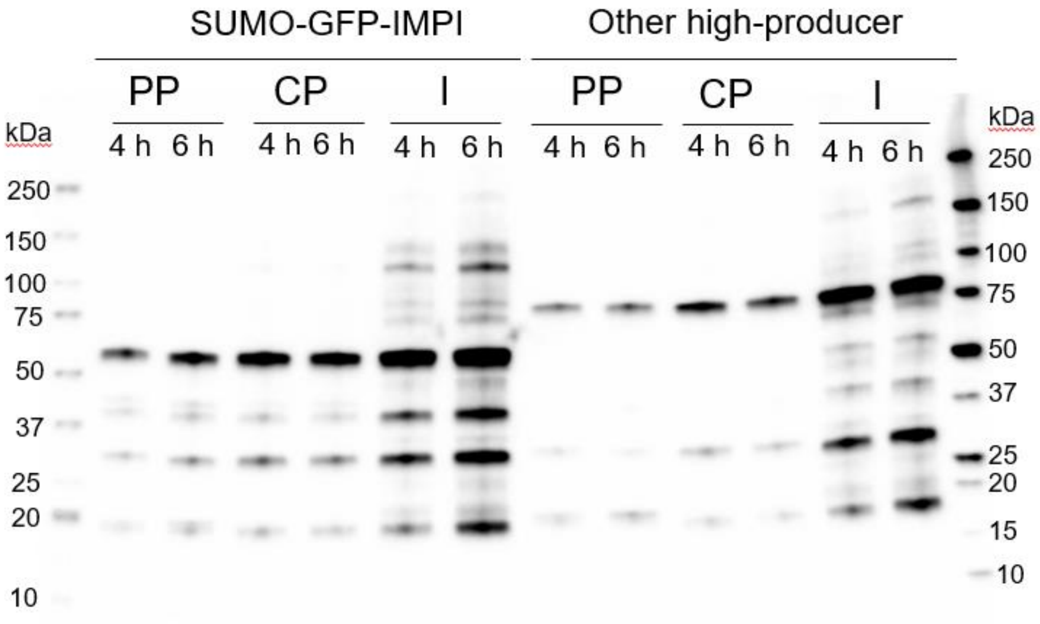

Supplementary Figure 7: Full Western Blot of high-producer SUMO-GFP-IMPI (left). Fig. 5 in the main article shows a cropped version of this image. Another high-producer not discussed in the main article is shown on the right site of the blot. PP: Periplasmic fraction; CP: Cytoplasmic fraction; I: Insoluble and membrane fraction. Standards left: Precision Plus Protein™ Western C Blotting Standard (BioRad); standard right: Precision Plus Protein™ Unstained Protein Standard, Strep-tagged (BioRad). Expected bands : left, SUMO-GFP-IMPI: 52,7 kDa; right, other high-producer: 77,2 kDa.

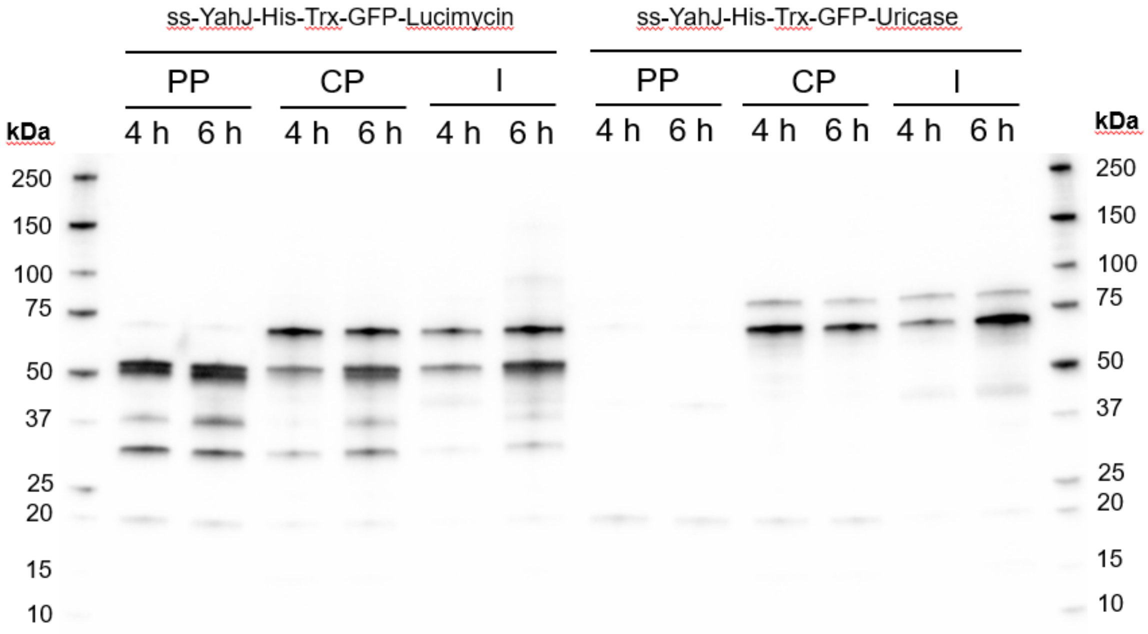

Supplementary Figure 8: Full Western Blot of high-producer ssYahJ-His-Trx-GFP fusion of Lucimycin (left) and Uricase (right). The left site of this blot is partially shown in in Fig. 6 of the main manuscript. PP: Periplasmic fraction; CP: Cytoplasmic fraction; I: Insoluble and membrane fraction. Standards left: Precision Plus Protein™ Western C Blotting Standard (BioRad); standard right: Precision Plus Protein™ Unstained Protein Standard, Strep-tagged (BioRad). Expected bands: left, ssYahJ-His-Trx-GFP-Lucimycin: 53,2 kDa; right, ssYahJ-His-Trx-GFP-Uricase: 83,2 kDa.

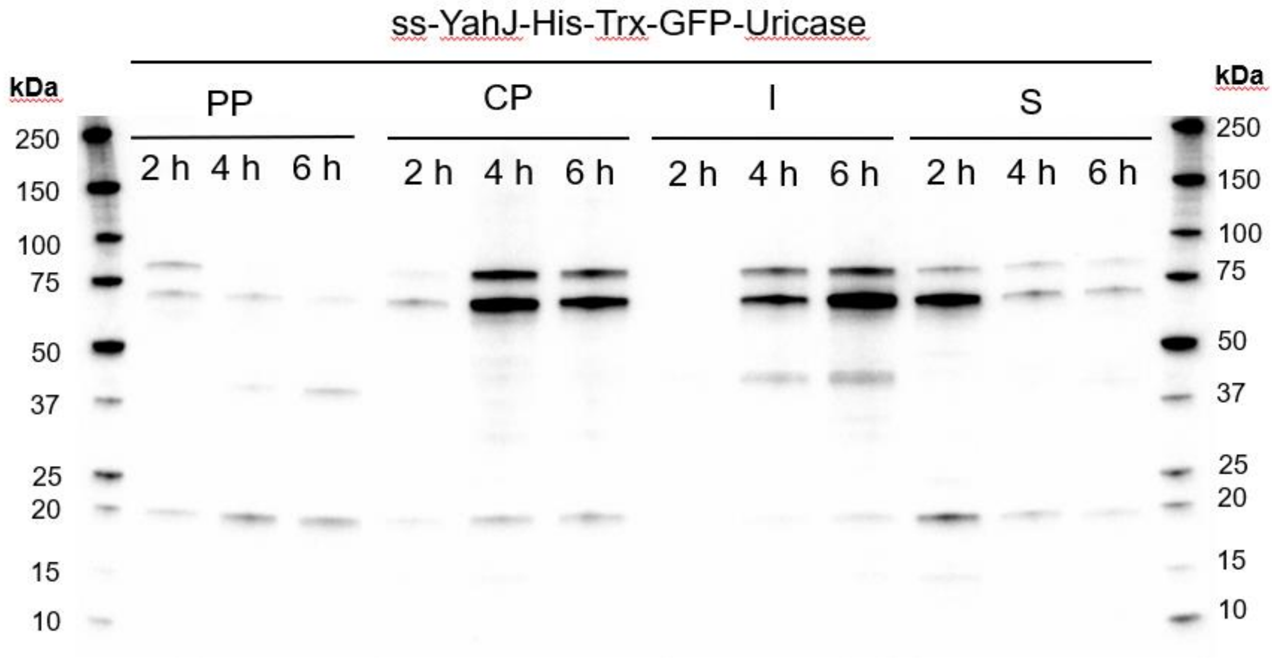

Supplementary Figure 9: Full Western Blot of high-producer ssYahJ-His-Trx-GFP-Uricase at all analysed time points. This blot is partially shown in in Fig. 7 of the main manuscript. PP: Periplasmic fraction; CP: Cytoplasmic fraction; I: Insoluble and membrane fraction; S: full soluble protein after BugBuster® extraction (diluted). Standards: Precision Plus Protein™ Unstained Protein Standard, Strep-tagged (BioRad). Expected bands: ssYahJ-His-Trx-GFP-Uricase: 83,2 kDa.

3.2 Agarose gels

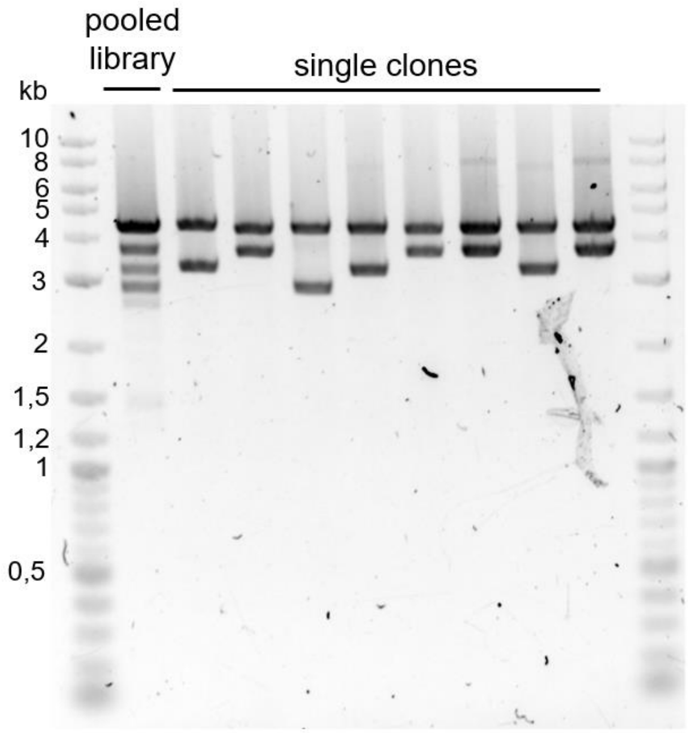

Supplementary Figure 10: BsaI-digestion of combinatorial expression library and single clones isolated from the library. The upper band represents the plasmid backbone and is expected at 4302 bp. The lower bands represent the inserts. Due to the size difference of the fusion tags for purification, the inserts differ in size. All sizes are present in the library, while single clones carry only one of the possible inserts. Marker: 2-Log DNA Ladder (NEB). A cropped version of this image is found in the main article in Fig. 1 B.

Literature

Espah Borujeni, Amin; Channarasappa, Anirudh S.; Salis, Howard M. (2014): Translation rate is controlled by coupled trade-offs between site accessibility, selective RNA unfolding and sliding at upstream standby sites. In: *Nucleic acids research* 42 (4), S. 2646–2659. DOI: 10.1093/nar/gkt1139.

Salis, Howard M.; Mirsky, Ethan A.; Voigt, Christopher A. (2009): Automated design of synthetic ribosome binding sites to control protein expression. In: *Nature biotechnology* 27 (10), S. 946–950. DOI: 10.1038/nbt.1568.
